# Early Identification of a Cytokine-Low Immune Phenotype in Suspected Sepsis Using a Point-of-Care Whole-Blood TNF-α Release Assay

**DOI:** 10.3390/medsci14040465

**Published:** 2026-08-08

**Authors:** Erika P. Plata-Menchaca, Berta Cisteró, Adrian Ceccato, Veronica Monforte, Queralt Caus-Capdevila, Aina Areny-Balaguero, Elena Campaña-Duel, Marta Camprubí-Rimblas, Gemma Goma-Fernandez, Carla Guijarro, Patricia Salom, Monica Lopez, Juan Tajan, Tiago Teles De Castro, Vanessa Roman, Joan Vieyra, Judit Cubedo, Eduard Guerrero, Emili Gené, Antonio Artigas, Enrique Hernández-Jiménez

**Affiliations:** 1 Medicine Department, Universitat Autònoma de Barcelona, Bellaterra, 08193 Barcelona, Spain; 2 Hamad Medical Corporation, Medical Intensive Care Unit Department, Doha P.O. Box 3050, Qatar; 3 Emergency Department, Hospital Universitari Parc Taulí, Institut d’Investigació i Innovació Parc Taulí (I3PT-CERCA), 08208 Sabadell, Spain; 4 Critical Care Center, Hospital Universitari Parc Taulí, Institut d’Investigació i Innovació Parc Taulí (I3PT-CERCA), Department of Medicine, Universitat Autònoma de Barcelona, 08208 Sabadell, Spain; 5 Centro de Investigación Biomédica en Red en Enfermedades Respiratorias (CIBERES), Instituto de Salud Carlos III, 28029 Madrid, Spain; 6 Department of Pharmacy and Pharmaceutical Technology and Physical Chemistry, University of Barcelona, 08028 Barcelona, Spain; 7 Loop Dx, StartUB!-University of Barcelona, 08028 Barcelona, Spain

**Keywords:** suspected sepsis, sepsis, host response, cytokine-low phenotype, TARA, TNF-α release assay, innate immune hyporesponsiveness, emergency department

## Abstract

Background/Objectives: Routine biomarkers and clinical scores do not directly assess functional immune responsiveness in suspected sepsis. We evaluated whether the TNF-α Release Assay (TARA), a whole-blood LPS-induced functional assay, is associated with an early cytokine-low immune phenotype in Emergency Department (ED) patients. Methods: In this prospective single-center cohort, adults with suspected sepsis and NEWS2 ≥ 3 were sampled at presentation, at 4 h and, when available, at 24 h. Immune phenotypes were defined independently of TARA by unsupervised k-means clustering of 12 circulating cytokines at 4 h, and a continuous Low-Response Cytokine Score (LRCS) was derived, with higher values indicating lower global cytokine concentrations. Results: Among 152 patients, three phenotypes were identified: C1 cytokine-low (*n* = 56), C2 inflammatory/intermediate (*n* = 63), and C3 IFN-γ/MCP-3/IL-17A-high (*n* = 33). Indicative TARA low-response status was independently associated with a higher LRCS after adjustment for clinical covariates and procalcitonin (PCT), both at presentation (β = 0.33; 95% CI, 0.14–0.53; *p* = 0.001) and at 4 h (β = 0.38; 95% CI, 0.19–0.57; *p* < 0.001). Indicative TARA low-response results at 4 h were most frequent in C1 and least frequent in C3 (75% vs. 45%). Exploratory clinical comparisons showed lower recorded early escalation in C1, whereas Sepsis-3 final diagnosis and in-hospital mortality were similar across phenotypes. Conclusions: A TARA low-response status was independently associated with an early circulating cytokine-low phenotype defined without reference to TARA or outcomes, providing information complementary to routine biomarkers and clinical severity scores. These exploratory findings support TARA as a complementary functional immune readout but require external validation and clinical-impact evaluation.

## 1. Introduction

Sepsis is a life-threatening syndrome caused by a dysregulated host response to infection, affecting an estimated 49 million people annually and responsible for approximately 11 million deaths each year [1,2]. Early recognition in the Emergency Department (ED) is particularly challenging because patients often present with non-specific symptoms, variable physiological deterioration and incomplete evidence of organ dysfunction [3,4]. This diagnostic uncertainty can delay antimicrobial therapy in patients with evolving sepsis while contributing to unnecessary antibiotic exposure in those with self-limited or non-bacterial conditions [5,6]. This distinction is clinically important because delays in administering appropriate antimicrobial therapy are associated with increased mortality in patients with severe sepsis and septic shock [7,8]. Current ED evaluation relies on clinical judgment, bedside severity scores and conventional biomarkers. NEWS2, qSOFA, SOFA and APACHE II support risk stratification but primarily capture physiological derangement or established organ dysfunction rather than the underlying host immune state. NEWS2 ≥ 3 identifies patients at elevated risk of deterioration and provides a pragmatic, clinically anchored inclusion threshold for ED sepsis research [9]. C-reactive protein, PCT and lactate are limited by variable sensitivity and specificity in heterogeneous populations with suspected sepsis [1,6,10], and pathogen-based diagnostics, while essential, do not provide immediate information on immune competence [11]. Early sepsis management therefore remains frequently guided by incomplete biological information. Sepsis is not a single biological entity but a heterogeneous syndrome composed of distinct host-response phenotypes [10,11]. Molecular and protein-based studies have identified subgroups with divergent inflammatory, endothelial, metabolic and immunosuppressive signatures that may differ in prognosis and response to therapy [12,13], yet whether biologically meaningful immune phenotypes can be identified earlier, at ED presentation, remains poorly characterized. Among the immune alterations observed in sepsis, impaired innate immune responsiveness has emerged as a clinically relevant mechanism, commonly characterized by reduced monocyte antigen presentation, lymphocyte dysfunction and blunted cytokine production after ex vivo stimulation [14,15]. Ex vivo LPS-induced TNF-α release provides a functional readout of innate immune competence and has been used as a model of endotoxin tolerance and immunoparalysis [16,17,18,19,20], but conventional cytokine release assays have historically been difficult to implement in acute care due to requirements for laboratory infrastructure and trained personnel [21]. Reduced whole-blood LPS-stimulated cytokine release, including that of TNF-α, has been associated with immune dysfunction and adverse clinical outcomes in critically ill and septic patients [22], a finding subsequently supported by systematic review evidence [23]. The TNF-α Release Assay (TARA) is a point-of-care whole-blood functional immune test that measures inducible TNF-α release after ex vivo LPS stimulation [24]. A reduced response provides a functional readout compatible with endotoxin tolerance state but does not by itself establish generalized immunoparalysis. Because TARA is performed on fresh whole blood within a timeframe compatible with ED workflows, it may provide complementary biological information to conventional biomarkers by directly interrogating immune responsiveness rather than measuring downstream inflammation or organ failure.

In this study, we investigated whether an indicative TARA low-response result identifies an early circulating cytokine-low immune phenotype in adults presenting to the ED with suspected sepsis and NEWS2 ≥ 3. Immune phenotypes were defined by unsupervised clustering of the standardized 12-cytokine matrix at 4 h, without including TARA results, routine biomarkers, microbiology or clinical outcomes in phenotype assignment. We hypothesized that an indicative TARA low-response result would be independently associated with an early cytokine-low immune phenotype, supporting biological concordance between reduced inducible TNF-α responsiveness and lower circulating cytokine concentrations not fully captured by routine biomarkers or clinical severity scores.

## 2. Materials and Methods

### 2.1. Study Design, Setting and Ethics

We conducted a prospective, single-center observational cohort study in the Emergency Department of Hospital Universitari Parc Taulí, Sabadell, Spain, enrolling consecutive adult patients between May 2022 and November 2024. Consecutive adult medical patients aged ≥18 years presenting with suspected sepsis at triage or during the Emergency Department stay and a National Early Warning Score 2 (NEWS2) ≥ 3 were eligible for inclusion. Patients evaluated for urgent or emergent surgical conditions, including trauma or acute surgical abdomen, were excluded. This study was exploratory and observational in design. No formal sample size calculation was performed. The analysis cohort of 152 patients was determined by the availability of sufficient cytokine data at 4 h during the study period. Baseline characteristics were compared between the 152 patients included in the 4 h cytokine analysis and the 21 patients who were not included. No statistically significant differences were observed in age, NEWS2, qSOFA, SOFA, shock or in-hospital mortality (Supplementary Table S1). All patients received standard clinical care according to local procedures and applicable sepsis management recommendations. The study was observational, and neither TARA results nor cytokine measurements were used to guide patient management. The study was conducted in accordance with the Declaration of Helsinki and applicable local regulations. Ethical approval was obtained from the institutional review board of Hospital Universitari Parc Taulí under protocol Septip: 2022/3004. The date of approval is 22 February 2022. Written informed consent was obtained from all participants or from their legally authorized representatives, as applicable.

### 2.2. Participants, Clinical Data and Sampling Time Points

Whole-blood samples were obtained at Emergency Department presentation, defined as 0 h, and again at 4 h. When available according to the study protocol and clinical workflow, an additional sample was collected at 24 h for longitudinal biological characterization.

Demographic characteristics, comorbidities, time from symptom onset to baseline sampling, baseline corticosteroid treatment, suspected infection focus, recorded microbiological findings, routine laboratory biomarkers, clinical severity scores, treatments and hospital outcomes were collected using standardized case report forms and electronic medical records. Severity scores included NEWS2, quick Sequential Organ Failure Assessment (qSOFA) and Sequential Organ Failure Assessment (SOFA), when available at each study time point. Clinical variables and outcomes were not used to define cytokine-derived immune phenotypes or to construct the Low-Response Cytokine Score.

### 2.3. TARA Functional Immune Assay

The TNF-α Release Assay (TARA; SeptiLoop^®^, Loop Diagnostics, Barcelona, Spain) is an ex vivo whole-blood functional assay that measures inducible TNF-α release after LPS stimulation, with results available within approximately 3 h of blood collection (assay procedure detailed in Supplementary Methods; Supplementary Figure S1). TARA results were classified according to the predefined assay interpretation as either indicative low-response, reflecting reduced LPS-induced TNF-α release compatible with impaired innate immune responsiveness, or preserved response, and were analyzed at presentation, 4 h and, when available, 24 h. An indicative low-response result was predefined as stimulated TNF-α release below 250 pg/mL [24] (limit of detection, LOD, see Supplementary Material), according to the validated assay interpretation.

#### 2.3.1. Plasma Cytokine Measurement

Plasma was obtained from lithium-heparin blood samples and stored in aliquots at −80 °C until analysis. Circulating concentrations of a 12-analyte cytokine panel were quantified using a custom human multiplex Luminex assay (Human ProcartaPlex Mix&Match 12-plex, Invitrogen, Thermo Fisher Scientific, Vienna, Austria), according to the manufacturer’s procedures (full panel listed in Supplementary Methods).

#### 2.3.2. Cytokine-Derived Immune Phenotype Definition

Cytokine-derived immune phenotypes were defined independently of TARA results, routine inflammatory biomarkers, clinical severity scores, microbiological findings and clinical outcomes, by unsupervised k-means clustering of the standardized 12-dimensional cytokine matrix measured at 4 h (clustering parameters detailed in Supplementary Methods). The 4 h cytokine matrix was selected as the single reference time point because it provided a standardized early post-presentation window in which circulating cytokines and TARA were evaluated contemporaneously. Defining phenotypes at one time point avoided generating separate, potentially non-comparable cluster assignments at each visit and avoided treating repeated observations from the same patient as independent data. Presentation and 24 h measurements were used only for longitudinal characterization according to the fixed 4 h phenotype assignment.

A three-cluster solution was retained for the primary analysis based on its biological interpretability and its suitability for evaluating a cytokine-low immune state alongside an alternative inflammatory phenotype. The four-cluster solution did not yield an additional biologically interpretable phenotype beyond the subdivision of the inflammatory/intermediate cluster and was therefore not retained for the primary analysis. K-means was selected as a transparent and reproducible exploratory approach for partitioning patients according to continuous, log-transformed and standardized cytokine concentrations in the original 12-dimensional feature space. Given the cohort size and number of variables, this approach limited model complexity and avoided the estimation of cluster-specific covariance matrices. K-means++ initialization, 100 random initializations and a fixed random seed were used to reduce sensitivity to local minima (Supplementary Figure S3).

The resulting phenotypes were interpreted from their mean standardized cytokine signatures: C1, cytokine-low phenotype: globally reduced circulating cytokine concentrations; C2, inflammatory/intermediate phenotype: enrichment for inflammatory and immunoregulatory mediators, including IL-6, IL-1RA and MCP-1; and C3, IFN/Th17-high phenotype: enrichment for IFN-γ, MCP-3 and IL-17A. Principal component analysis (PCA) was applied to the standardized 4 h cytokine matrix for visualization only. PCA coordinates were not used for clustering, phenotype assignment, score construction or statistical testing.

### 2.4. Primary and Secondary Analyses

The primary analysis evaluated whether indicative an low-response TARA measured at 4 h was associated with higher Low-Response Cytokine Score (LRCS) 4 h values, using ordinary least squares regression with heteroskedasticity-consistent HC3 robust standard errors, analyzed unadjusted, adjusted for clinical covariates, and additionally adjusted for log10(PCT + 1). A key secondary analysis applied the same model structure to TARA measured at presentation. To complement the continuous LRCS analysis, the distribution of indicative TARA low-response results was compared across C1, C2 and C3 at presentation, 4 h and 24 h, and adjusted logistic regression was used to estimate the association between TARA status and predefined phenotype-level contrasts, including indicative TARA low-response results with C1 membership and a preserved TARA response with C3 membership.

Longitudinal cytokine trajectories, routine inflammatory biomarkers, hematological variables, and clinical severity scores were analyzed according to fixed 4 h phenotype membership. Cytokine measurements at 0 h and 24 h were used only for longitudinal characterization and did not contribute to phenotype assignment.

#### 2.4.1. Clinical, Microbiological and Outcome Analyses

Clinical and microbiological characteristics were compared descriptively across cytokine-derived immune phenotypes. Variables included suspected infection focus, documented organism, positive blood culture, shock, vasopressor use, intensive care unit admission, in-hospital death and hospital length of stay. Microbiological documentation was derived from the recorded organism field. The absence of a documented organism was interpreted as the absence of a recorded organism, not as confirmed microbiological negativity.

Because the cytokine-low phenotype was enriched for an indicative TARA low-response but did not show the highest frequency of acute clinical escalation, an exploratory mortality pathway analysis classified in-hospital outcomes as alive or discharged, death after recorded ICU admission, or death without recorded ICU admission. ICU eligibility, treatment limitation decisions, goals of care discussions, and palliative care status were not available; therefore, death without recorded ICU admission should not be interpreted as evidence of inadequate escalation of care.

#### 2.4.2. Statistical Analysis

The prospective clinical protocol, eligibility criteria, sampling schedule, and assay procedures were defined before enrollment. However, LRCS construction, isolated-value imputation, and clustering were not covered by a preregistered statistical analysis plan and are therefore considered exploratory. Formal normality testing was not used as a decision rule for statistical-test selection. Because cytokines and several clinical biomarkers showed skewed distributions, descriptive data were summarized using medians and interquartile ranges, and group comparisons were performed using non-parametric tests. The principal analysis evaluated the association between indicative TARA low-response status at 4 h and LRCS-4h. The corresponding analysis using TARA at presentation was considered a key secondary analysis. Phenotype-level regressions and comparisons involving individual cytokines, longitudinal measurements, routine biomarkers, hematological variables, microbiological characteristics and clinical outcomes were considered secondary or exploratory. Because LRCS-4h was standardized to a mean of 0 and a standard deviation of 1, regression coefficients for binary predictors were interpreted as adjusted mean differences expressed in LRCS standard-deviation units. Complete-case analysis was used for each regression model. Clinically adjusted models included 148 patients with paired TARA and LRCS-4h data, whereas models additionally incorporating PCT included 141 patients. Models excluding PCT retained the larger paired cohort and were used to assess consistency under an alternative covariate specification but were not considered a formal correction for potentially informative PCT missingness. No adjustment for multiplicity was applied to secondary or exploratory analyses. Their *p* values and confidence intervals are therefore nominal and should be interpreted descriptively and as hypothesis-generating rather than as confirmatory evidence.

Analyses were performed using Python 3.13.5. (Python Software Foundation, Wilmington, DE, USA). Continuous variables are summarized as medians (IQR) or means (SE), as appropriate. Comparisons across immune phenotypes used Kruskal–Wallis tests for continuous variables and Fisher exact or chi-square tests for categorical variables; pairwise TARA-group comparisons used two-sided Mann–Whitney U tests. Regression analyses were performed using ordinary least squares models with HC3 robust standard errors. All tests were two-sided, and *p* values below 0.05 were considered statistically significant. Secondary and exploratory analyses were not adjusted for multiplicity and should be interpreted as hypothesis-generating. Sensitivity analyses are described in the Supplementary Methods.

## 3. Results

### 3.1. Study Population and Definition of Early Cytokine-Derived Immune Phenotypes

A total of 173 patients had available cytokine-clinical data. Of these, 152 patients had sufficient cytokine data at 4 h to calculate the LRCS and to define cytokine-derived immune phenotypes. Patient availability for paired TARA analyses and for the fully adjusted regression models is summarized in Supplementary Figure S4.

Among these 152 patients, 148 had paired LRCS-4h and TARA results at presentation, and 148 had paired LRCS-4h and TARA results at 4 h. According to the patient-flow diagram, complete-case cohorts for the fully adjusted PCT models included 141 patients for both the presentation TARA and 4 h TARA analyses.

Direct k-means clustering of the standardized 12-cytokine matrix measured at 4 h identified three biologically interpretable immune phenotypes: C1, a cytokine-low phenotype (*n* = 56); C2, an inflammatory/intermediate phenotype (*n* = 63); and C3, an IFN-γ/MCP-3/IL-17A-high phenotype (*n* = 33). The three-cluster solution achieved the highest silhouette score among the candidate solutions evaluated (Supplementary Figure S2) and was retained for the primary analyses, as the four-cluster solution did not yield an additional biologically distinct phenotype beyond the subdivision of the inflammatory/intermediate cluster (Supplementary Figure S2). Bootstrap internal validation supported the principal partition, with a median adjusted Rand index of 0.755 and mean cluster-specific Jaccard similarities of 0.844, 0.815 and 0.739 for C1, C2 and C3, respectively (Supplementary Figure S3). For completeness, the alternative two-cluster solution is presented in Supplementary Table S2 and Supplementary Figure S6.

Baseline clinical characteristics according to immune phenotype are presented in Table 1. Overall, the three phenotypes were comparable for several baseline characteristics, including age, sex distribution, clinical diagnosis of sepsis, suspected infection focus, PCT, CRP and qSOFA. Differences were observed in selected variables, including comorbidity burden, lactate, NEWS2 and neutrophil-to-lymphocyte ratio, indicating that the cytokine-derived phenotypes were associated with distinct but partially overlapping clinical presentations.

### 3.2. TARA Low-Response Status Is Independently Associated with the Early Cytokine-Low Phenotype

**Figure 1:**
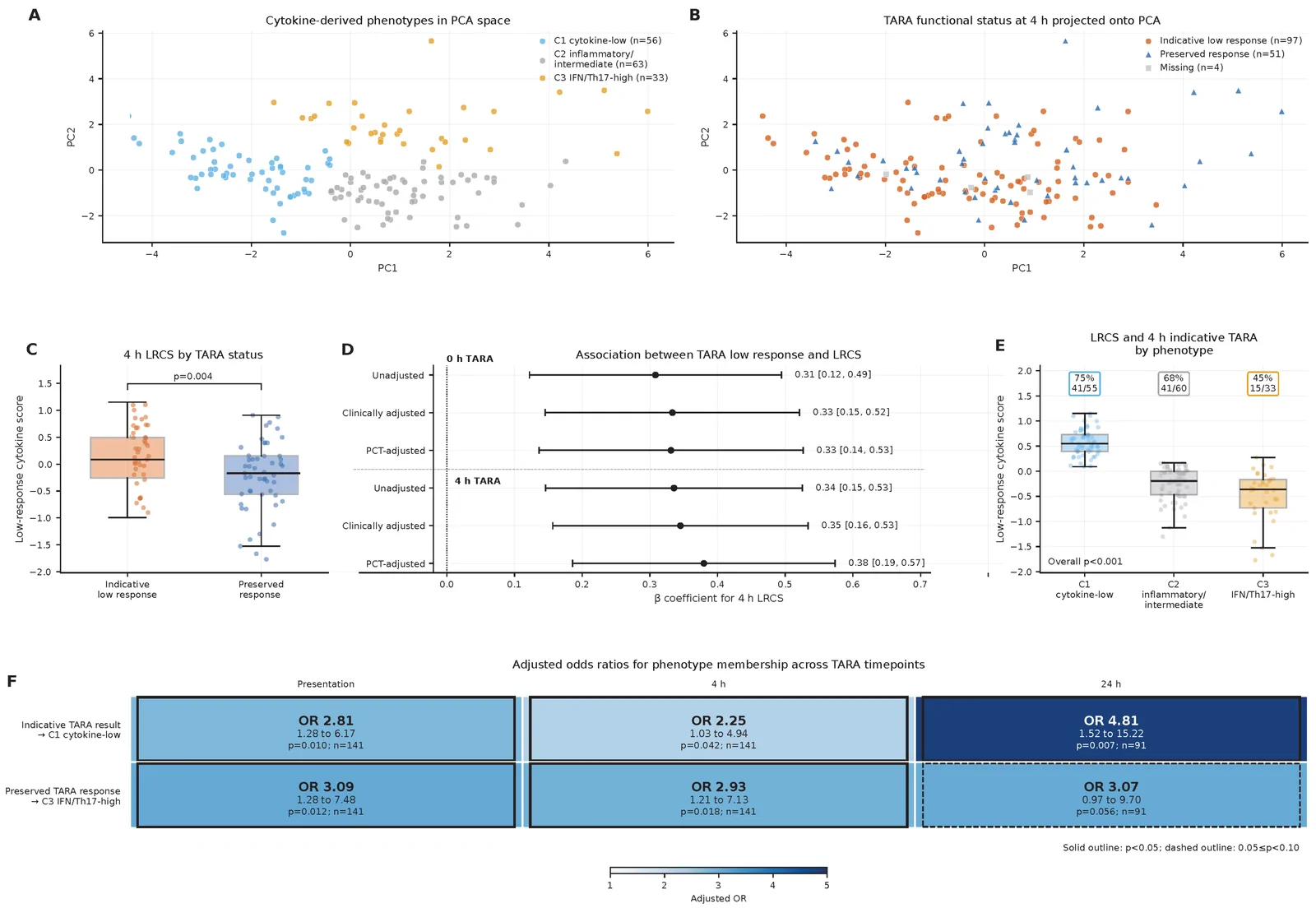
TARA functional status is associated with cytokine-derived immune phenotypes. Immune phenotypes were defined in 152 Emergency Department patients by k-means clustering of the standardized 12-cytokine matrix at 4 h: C1 cytokine-low (*n* = 56), C2 inflammatory/intermediate (*n* = 63), and C3 IFN/Th17-high (*n* = 33). (**A**), PCA visualization of the three phenotypes; crosses indicate cluster centroids. PCA was used only for visualization. (**B**), TARA status at 4 h projected onto the same PCA space: indicative low-response (*n* = 97), preserved response (*n* = 51), or missing result (*n* = 4). (**C**), LRCS-4h according to TARA status at 4 h; higher values indicate lower overall cytokine concentrations. The *p* value was calculated using a two-sided Mann–Whitney U test. (**D**), regression coefficients for the association of indicative TARA low-response status at presentation or 4 h with LRCS-4h. Points show β coefficients in LRCS standard-deviation units, and bars show 95% confidence intervals. Clinically adjusted models included age, Charlson comorbidity index, baseline NEWS2, symptom duration, and baseline corticosteroid treatment; PCT-adjusted models additionally included log10(PCT + 1). (**E**), LRCS-4h and the proportion of indicative TARA low-response results across phenotypes; the *p* value represents the global Kruskal–Wallis comparison. (**F**), adjusted odds ratios for the association of indicative TARA low-response status with C1 membership and preserved TARA status with C3 membership. Models included age, sex, Charlson comorbidity index, baseline SOFA, and log10(PCT + 1). Solid outlines indicate *p* < 0.05; dashed outlines indicate 0.05 ≤ *p* < 0.10. LRCS, Low-Response Cytokine Score; NEWS2, National Early Warning Score 2; PCA, principal component analysis; PCT, pro-calcitonin; SOFA, Sequential Organ Failure Assessment; TARA, TNF-α Release Assay.

Visualization of the exploratory three-cluster solution showed a clear organization of the three cytokine-derived immune phenotypes in principal component space, with C1 representing the broad cytokine-low state, C2 an inflammatory/intermediate state, and C3 an IFN/Th17-high state (Figure 1A). When TARA results measured at 4 h were projected onto the same space, indicative low-response results were enriched toward the cytokine-low phenotype, although they were not restricted to this cluster, indicating that TARA captures a functional low-response state that partly overlaps with, but does not fully recapitulate, the circulating cytokine phenotypes (Figure 1B). Consistent with this distribution, patients with an indicative TARA low-response result had higher 4 h LRCS values than patients with a preserved TARA response (Figure 1C). In regression analyses, an indicative TARA low-response status remained independently associated with higher LRCS values after adjustment for clinical covariates and PCT. This association was observed for TARA measured at presentation (β = 0.33 standard deviation units; 95% CI, 0.14 to 0.53; *p* = 0.001) and for TARA measured at 4 h (β = 0.38; 95% CI, 0.19 to 0.57; *p* < 0.001) (Figure 1D). LRCS values showed a marked biological gradient across the three cytokine-derived phenotypes, with the highest values in C1, intermediate values in C2 and the lowest values in C3. Indicative TARA low-response results at 4 h followed the same direction, being most frequent in C1 and least frequent in C3 (75% in C1, 68% in C2 and 45% in C3; Figure 1E). For completeness, the alternative two-cluster solution is presented in Supplementary Figure S6.

Finally, secondary adjusted logistic regression models supported the directional relationship between TARA functional status and phenotype membership across time points (Figure 1F). Indicative TARA low-response was independently associated with increased odds of belonging to the C1 cytokine-low phenotype when measured at presentation (adjusted OR 2.81; 95% CI, 1.28 to 6.17; *p* = 0.010), at 4 h (adjusted OR 2.25; 95% CI, 1.03 to 4.94; *p* = 0.042) and at 24 h (adjusted OR 4.81; 95% CI, 1.52 to 15.22; *p* = 0.007). Conversely, a preserved TARA response was associated with increased odds of belonging to the C3 IFN/Th17-high phenotype at presentation (adjusted OR 3.09; 95% CI, 1.28 to 7.48; *p* = 0.012) and at 4 h (adjusted OR 2.93; 95% CI, 1.21 to 7.13; *p* = 0.018), with a similar trend at 24 h (adjusted OR 3.07; 95% CI, 0.97 to 9.70; *p* = 0.056). Together, these findings support that a TARA low-response identifies a functional immune state aligned with a cytokine-low phenotype, whereas a preserved TARA response is preferentially associated with an IFN/Th17-high inflammatory phenotype. These supportive analyses were directionally consistent with the principal continuous LRCS analysis, although they were exploratory and were not adjusted for multiplicity.

### 3.3. Cytokine-Derived Immune Phenotypes Show Longitudinal Biological Coherence

**Figure 2:**
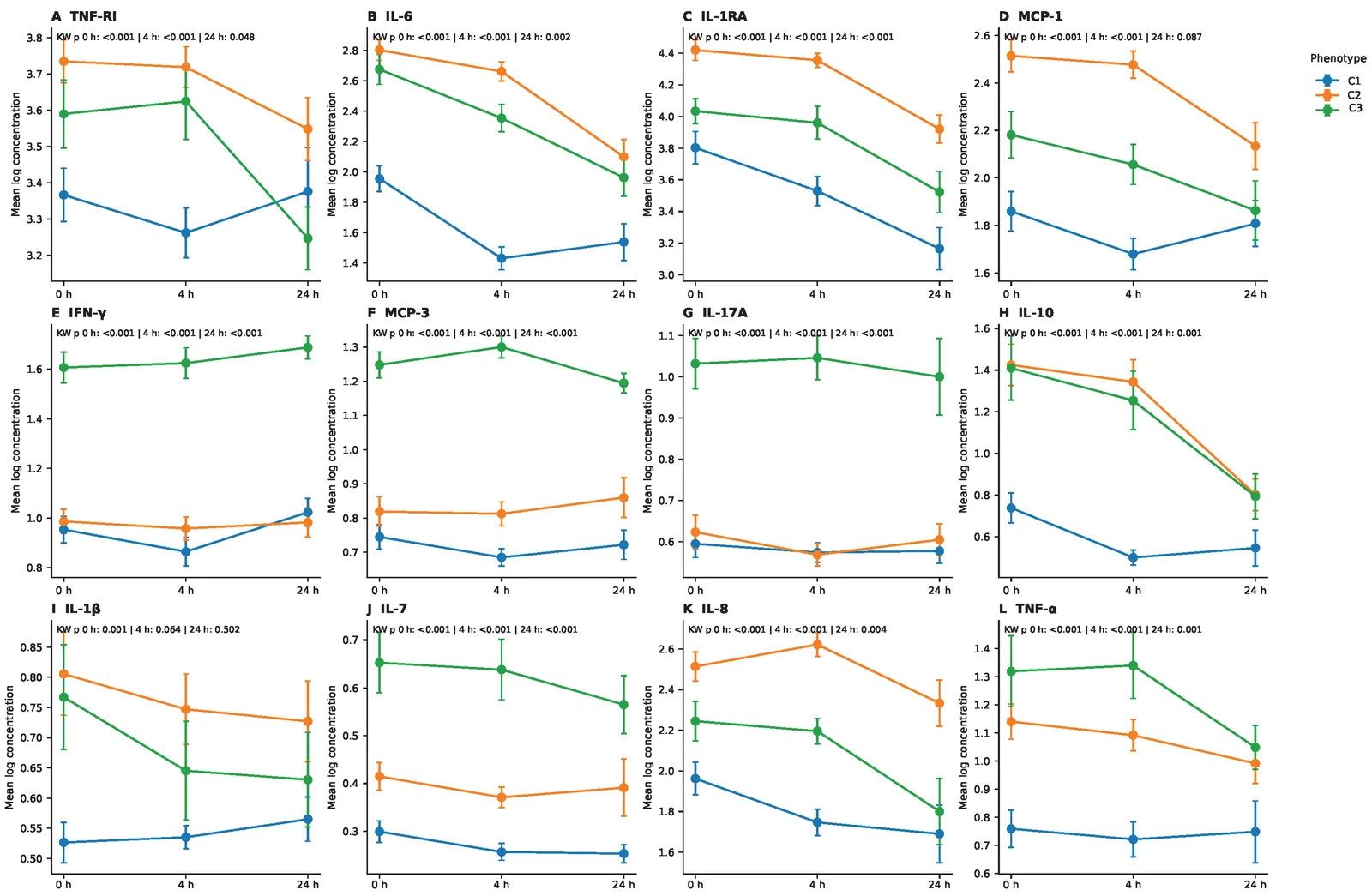
Longitudinal cytokine trajectories across 4 h-defined immune phenotypes. Circulating cytokine concentrations at presentation, 4 h, and 24 h according to phenotypes defined from the 4 h cytokine matrix: C1 cytokine-low, C2 inflammatory/intermediate, and C3 IFN/Th17-high. (**A**), TNF-RI; (**B**–**D**), IL-6, IL-1RA, and MCP-1; (**E**–**G**), IFN-γ, MCP-3, and IL-17A; (**H**–**L**), IL-10, IL-1β, IL-7, IL-8, and TNF-α. Phenotype assignment was fixed for longitudinal analyses. Cytokine concentrations were log10(concentration + 1)-transformed. Points represent means and error bars represent the standard error of the mean. Global *p* values were calculated using Kruskal–Wallis tests. Comparisons at 4 h are descriptive because these measurements were used to derive the phenotypes. No multiplicity adjustment was applied.

To assess whether the phenotypes defined at 4 h represented coherent biological states over time, cytokine measurements obtained at presentation and at 24 h were analyzed according to the fixed 4 h phenotype assignment. Longitudinal cytokine trajectories differentiated the three fixed 4 h immune phenotypes across the 12 analytes evaluated (Figure 2A–L). C1 generally showed lower circulating cytokine concentrations, C2 showed higher TNF-RI, IL-6, IL-1RA, MCP-1, IL-10 and IL-8 concentrations, and C3 showed higher IFN-γ, MCP-3, IL-17A, IL-7 and TNF-α concentrations. The remaining cytokines provided additional biological characterization of these states and confirmed that C1 represented a broadly attenuated circulating cytokine profile rather than a reduction limited to a single mediator. Importantly, phenotype membership was determined exclusively from the 4 h cytokine matrix. Earlier and later measurements were used only for longitudinal characterization and did not contribute to phenotype assignment.

### 3.4. Routine Biomarkers, Severity Scores and Hematological Variables Do Not Fully Reproduce the Cytokine-Low Phenotype

**Figure 3:**
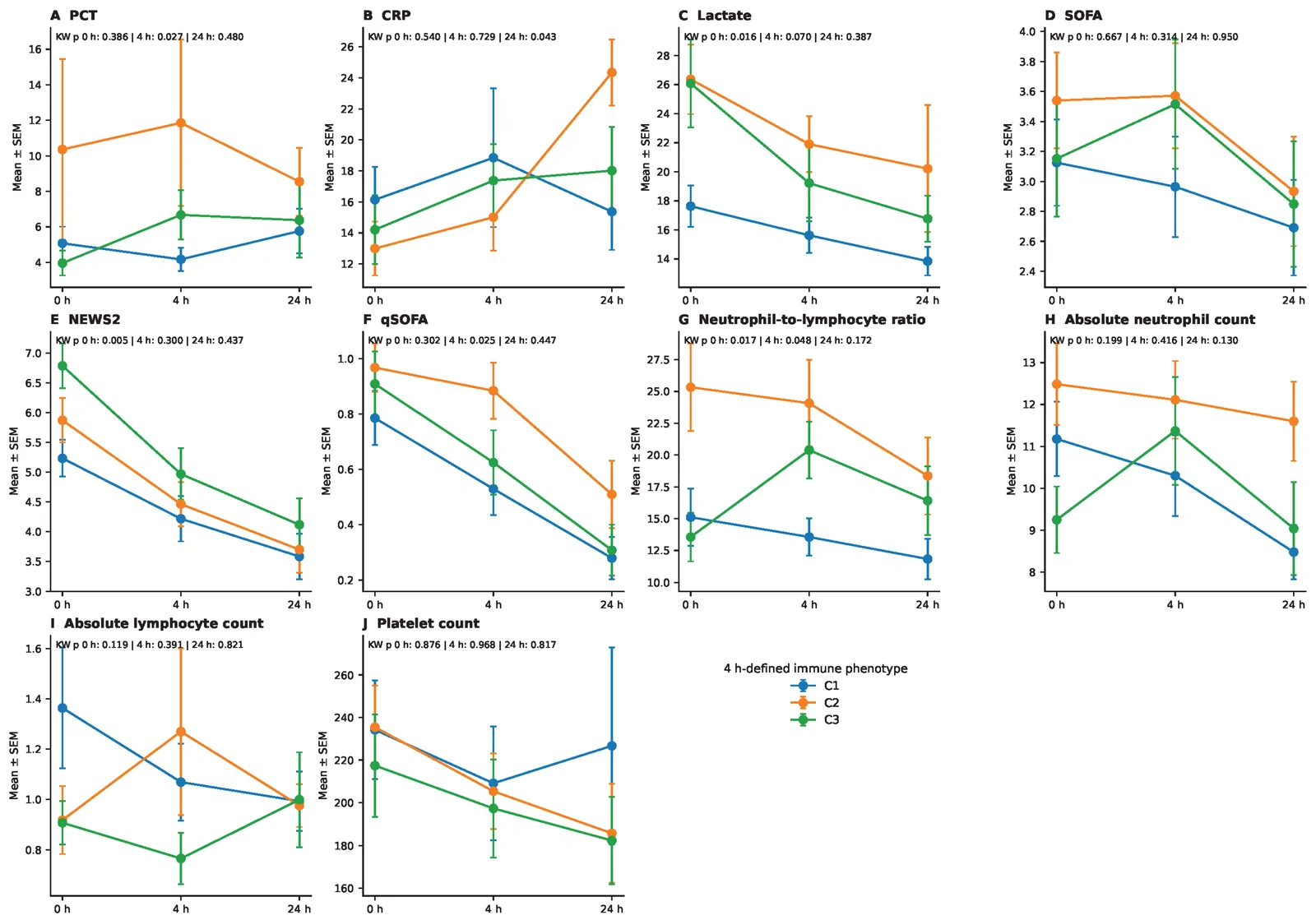
Routine biomarkers, severity scores, and hematological variables across immune phenotypes. Longitudinal profiles according to the 4 h-defined phenotypes C1 cytokine-low, C2 inflammatory/intermediate, and C3 IFN/Th17-high. (**A**–**C**), PCT, CRP, and lactate; (**D**–**F**), SOFA, NEWS2, and qSOFA; (**G**–**J**), neutrophil-to-lymphocyte ratio, absolute neutrophil count, absolute lymphocyte count, and platelet count. Phenotype assignment was fixed for all time points. Points represent means and error bars represent the standard error of the mean. Global *p* values were calculated using Kruskal–Wallis tests. NLR was calculated as the absolute neutrophil count divided by the absolute lymphocyte count; observations with zero lymphocytes were treated as missing. CRP, C-reactive protein; NEWS2, National Early Warning Score 2; NLR, neutrophil-to-lymphocyte ratio; PCT, procalcitonin; qSOFA, quick Sequential Organ Failure Assessment; SOFA, Sequential Organ Failure Assessment.

Routine inflammatory biomarkers and bedside severity scores showed phenotype-associated differences at selected time points, but these patterns only partially overlapped with the cytokine-derived immune phenotypes. Among routine inflammatory markers, PCT differed across phenotypes at 4 h (*p* = 0.027), CRP differed at 24 h (*p* = 0.043), and lactate differed at presentation (*p* = 0.016). However, these differences were time-dependent and did not reproduce the broad low-cytokine pattern that defined C1 (Figure 3A–C).

Clinical severity scores also showed selective rather than consistent phenotype-associated differences. NEWS2 differed across phenotypes at presentation (*p* = 0.005), whereas qSOFA differed at 4 h (*p* = 0.025). In contrast, SOFA did not differ significantly across phenotypes at any time point. Importantly, these score trajectories did not identify C1 as the phenotype with the greatest overt clinical severity, despite its cytokine-low profile (Figure 3D–F).

Exploratory hematological analyses showed that neutrophil-to-lymphocyte ratio differed across phenotypes at presentation (*p* = 0.017) and at 4 h (*p* = 0.048), whereas absolute neutrophil count, absolute lymphocyte count and platelet count did not show consistent significant differences across time points (Figure 3G–J). These findings indicate that hematological indices may capture selected inflammatory or cellular features but do not fully explain the cytokine-low phenotype.

Together, these results suggest that the cytokine-low dimension captured by the LRCS and associated with TARA reflects a biological dimension that is complementary to, rather than interchangeable with, routine inflammatory biomarkers, hematological measures, and early clinical severity scores.

### 3.5. Clinical Presentation, Microbiological Context and Exploratory Hospital Outcomes Across Immune Phenotypes

**Figure 4:**
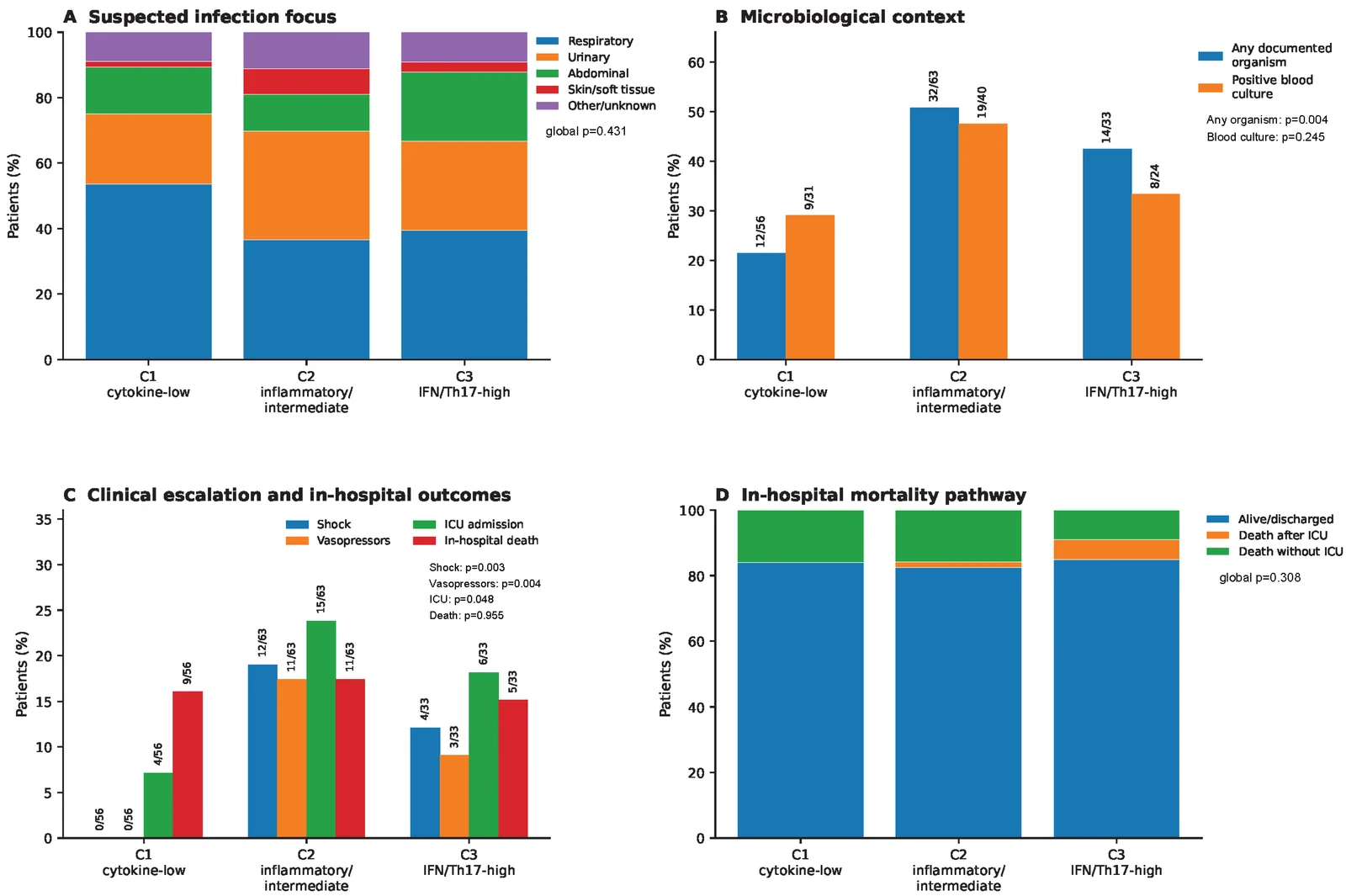
Clinical, microbiological, and exploratory outcome characteristics across immune phenotypes. Clinical characteristics are shown for C1 cytokine-low (*n* = 56), C2 inflammatory/intermediate (*n* = 63), and C3 IFN/Th17-high (*n* = 33). (**A**) Suspected infection focus. (**B**) Documented organisms and positive blood cultures. Positive blood-culture proportions were calculated among patients with available results; absence of a documented organism does not indicate confirmed microbiological negativity. (**C**) Shock, vasopressor use, ICU admission, and in-hospital death. (**D**) Exploratory mortality pathway classified as alive or discharged, death after recorded ICU admission, or death without recorded ICU admission. Fractions indicate events over the applicable denominator. Global *p* values were calculated using chi-square or Fisher’s exact tests, as appropriate. Outcome analyses were exploratory. Information on ICU eligibility, treatment limitations, goals of care, and palliative status was unavailable; death without recorded ICU admission should therefore not be interpreted as inadequate escalation of care. ICU, intensive care unit.

The distribution of suspected infection foci was broadly similar across the three cytokine-derived immune phenotypes (global *p* = 0.431; Figure 4A). Microbiological documentation differed across phenotypes. Any documented organism was less frequent in the cytokine-low C1 phenotype than in C2 or C3, being recorded in 12 of 56 C1 patients (21.4%), 32 of 63 C2 patients (50.8%) and 14 of 33 C3 patients (42.4%) (*p* = 0.004; Figure 4B). Among patients with available blood culture results, positive blood cultures were present in 9 of 31 C1 patients (29.0%), 19 of 40 C2 patients (47.5%) and 8 of 24 C3 patients (33.3%) (*p* = 0.245; Figure 4B). Because microbiological documentation was derived from the recorded organism field, the absence of a documented organism should not be interpreted as confirmed microbiological negativity. Exploratory stratification by documented pathogen Gram status included 40 patients with Gram-negative organisms and 13 with Gram-positive organisms. No clear differences were observed in indicative TARA low-response prevalence at presentation or 4 h, LRCS-4h, circulating TNF-α concentrations, or cytokine-derived phenotype distribution (Supplementary Table S3). These comparisons were limited by the small Gram-positive subgroup.

Clinical escalation patterns also differed across phenotypes. Despite being enriched in indicative TARA low-response results, C1 showed no recorded shock (0/56, 0%) and no recorded vasopressor use (0/56, 0%), with ICU admission recorded in only 4 of 56 patients (7.1%). By contrast, shock occurred in 12 of 63 C2 patients (19.0%) and 4 of 33 C3 patients (12.1%), vasopressor use in 11 of 63 C2 patients (17.5%) and 3 of 33 C3 patients (9.1%), and ICU admission in 15 of 63 C2 patients (23.8%) and 6 of 33 C3 patients (18.2%). Differences across phenotypes were significant for shock (*p* = 0.003), vasopressor use (*p* = 0.004) and ICU admission (*p* = 0.048; Figure 4C). In contrast, in-hospital mortality was similar across phenotypes, occurring in 9 of 56 C1 patients (16.1%), 11 of 63 C2 patients (17.5%) and 5 of 33 C3 patients (15.2%) (*p* = 0.955; Figure 4C). This dissociation between reduced ex vivo LPS-induced TNF-α responsiveness, lower early clinical escalation, and comparable mortality suggests that the cytokine-low immune state is not equivalent to overt acute clinical instability.

Exploratory analysis of the mortality pathway showed that all recorded deaths in C1 occurred without recorded ICU admission (9/9 deaths), whereas deaths after ICU admission were observed in C2 and C3 (Figure 4D). Although the global comparison of mortality pathway distribution did not reach statistical significance (*p* = 0.308), this pattern suggests that the cytokine-low phenotype may identify a subgroup of patients with biological vulnerability that is not fully reflected by early shock, vasopressor requirement or ICU admission. One patient experienced a terminal event before the 4 h landmark and was excluded from these analyses. Exploratory cumulative incidence analyses treating in-hospital death and discharge alive as competing events are presented in Supplementary Figure S5. Clinical outcome comparisons were exploratory. C1 showed less recorded shock, vasopressor use, and ICU admission, whereas in-hospital mortality was similar across phenotypes. These observations should not be interpreted as evidence of prognostic utility because the study was not powered for clinical outcomes, and information on treatment limitations or goals of care was unavailable.

## 4. Discussion

In this prospective ED cohort of patients with suspected sepsis and NEWS2 ≥ 3, we identified three exploratory cytokine-derived phenotypes with partially distinct biological and functional profiles. The principal finding is that an indicative TARA low-response result was independently associated with an early circulating cytokine-low phenotype, both at presentation and at 4 h, and remained significant after adjustment for clinical covariates and PCT. Because the cytokine-low dimension was defined independently of TARA, routine biomarkers, microbiology and clinical outcomes, these findings support TARA as a functional readout of reduced ex vivo LPS-induced TNF-α responsiveness associated with an early circulating cytokine-low dimension. This is biologically meaningful because CRP, PCT and lactate primarily reflect inflammatory activity, infection probability or physiological consequences of acute illness, and NEWS2, qSOFA and SOFA describe clinical deterioration, whereas TARA directly interrogates the capacity of whole blood to release TNF-α after a standardized ex vivo LPS challenge, a readout compatible with an endotoxin tolerance state.

The continuous LRCS strengthens this interpretation by quantifying cytokine attenuation across all 12 mediators rather than relying on discrete clustering. Patients with indicative TARA low-response results had higher LRCS values whether TARA was measured at presentation or 4 h. However, indicative results were not restricted to C1 and remained frequent in C2, indicating that the functional low-response and the circulating cytokine phenotype are related but non-identical dimensions: some patients simultaneously display circulating inflammation and reduced inducible responsiveness, consistent with the coexistence of inflammation and immune dysfunction during acute infection.

### 4.1. Biological Interpretation and Established Endotypes

The three clusters captured distinct host-response states: C1, globally low cytokines; C2, an inflammatory/immunoregulatory profile enriched for IL-1RA, MCP-1, IL-6 and IL-8; and C3, an IFN-γ/MCP-3/IL-17A-high profile. A Sepsis-3 diagnosis was similarly distributed across clusters in this cohort, although the study was not powered to establish equivalence between phenotypes. These findings are consistent with sepsis as a heterogeneous syndrome in which the Sepsis-3 definition does not specify the type of immune dysregulation present [1]. Such heterogeneity may contribute both to difficult early ED recognition and to the limited success of non-stratified therapies. Against a major global burden [2,3,4,5], early recognition is critical because delayed antimicrobial therapy worsens outcomes in severe sepsis and septic shock [6,7,8], yet early presentations are often non-specific and organ dysfunction may be absent or evolving [25,26]. Current bedside scores capture physiological deterioration rather than the immune state [9,25,27]; PCT, CRP, lactate and leukocyte indices have variable diagnostic performance [10,12,13]; pathogen-based diagnostics do not measure the host response [11]; and emerging host-response and AI-based tests integrate biological signals [14,28,29] but rarely provide a direct functional measure of innate competence.

C1 showed the highest proportion of indicative TARA low-response results at all time points and the most persistent indicative low-response trajectories; the combination of low circulating cytokines and persistently impaired inducible TNF-α release is compatible with endotoxin tolerance [16,30,31]. Notably, C1 was not the cluster with the greatest clinical severity, shock or ICU admission, indicating that functional immunocompromise can be present early even in patients who are not the most clinically unstable, immune and organ dysfunction are related but not interchangeable dimensions. The C1 cytokine-low phenotype should not be interpreted as established immunoparalysis. Rather, it represents a circulating cytokine-low state independently associated with reduced ex vivo LPS-induced TNF-α release. TARA provides a functional readout of one component of innate immune responsiveness, but it does not characterize the full immune state. In the absence of monocyte HLA-DR measurements, immune-cell phenotyping, lymphocyte-function testing, transcriptomics or additional functional stimulation assays, we cannot determine whether C1 reflects generalized immune suppression, altered leukocyte composition, transient regulatory adaptation or another host-response program. The present findings therefore support biological concordance between reduced circulating cytokine concentrations and reduced inducible TNF-α responsiveness.

C2, the largest cluster, was enriched for IL-1RA, MCP-1, IL-6, IL-8 and TNF-RI, with higher rates of shock and ICU admission and a trend toward more frequent blood-culture positivity, compatible with a mixed inflammatory/immunoregulatory response in which systemic inflammation coexists with functional impairment. This aligns with models emphasizing simultaneous inflammatory, endothelial, metabolic and suppressive pathways [16,17,32] and with work linking reduced ex vivo LPS-stimulated cytokine release and monocyte HLA-DR downregulation to adverse outcomes and secondary infection [21,24,27,33,34,35]. Direct comparisons between stimulated TNF-α release and mHLA-DR have shown that both markers carry complementary prognostic information in critically ill patients [36,37]; TARA may thus offer a practical functional readout complementing mHLA-DR.

By contrast, C3 was enriched for IFN-γ, MCP-3, IL-17A, IL-7 and TNF-α and had the lowest proportion of indicative TARA low-response results. Although limited by small event numbers, this raises the hypothesis that the prognostic meaning of impaired inducible TNF-α release depends on the broader cytokine context: in an IFN-γ/MCP-3/IL-17A-dominant state, a TARA result may reflect a distinct immune program rather than preserved innate competence. TARA should therefore not be interpreted as a context-free binary marker.

The present phenotypes should therefore be considered exploratory protein-level representations of early host-response heterogeneity rather than novel molecular endotypes or validated surrogates of existing classifications. Their correspondence with SRS, MARS, inflammopathic, adaptive, coagulopathic or SENECA phenotypes will require studies integrating TARA and cytokine measurements with transcriptomics, leukocyte phenotyping and established endotype classifiers.

### 4.2. Microbiological Context and Longitudinal Interpretation

The exploratory microbiological findings provided additional context for the cytokine-derived phenotypes. C2 showed a numerically higher proportion of positive blood cultures, although the global comparison was not statistically significant and was not adjusted for multiplicity; while C1 was enriched for respiratory infections, broadly consistent with recent protein-based phenotyping of ED patients with suspected sepsis, where host-response clusters showed distinct pathogen patterns, organ dysfunction and outcomes [38]. Our study extends this framework by adding a functional whole-blood immune assay and repeated early time points.

The cytokine-derived phenotypes should not be considered direct equivalents of the SENECA (Sepsis Endotyping in Emergency Care) α, β, γ and δ clinical phenotypes; rather, they provide a functional immune layer that partially overlaps with, but is biologically distinct from, EHR-derived clinical phenotypes. In particular, the C1 cytokine-low phenotype enriched for indicative TARA low-response results may represent an immunosuppressed β-like state, whereas the inflammatory and IFN-γ/MCP-3/IL-17A-high profiles refine the broad SENECA γ inflammatory phenotype into functionally distinct immune-response patterns [39]. The persistence of several defining cytokine differences at presentation and 24 h supports short-term biological coherence of the 4 h phenotype assignment, although it does not establish stable longitudinal endotypes.

Defining cytokine differences were maintained across 0 h, 4 h and 24 h for multiple analytes (IL-6, IL-1RA, IL-8, MCP-1, TNF-α, TNF-RI, IFN-γ, MCP-3 and IL-17A), indicating that the clusters represent short-term immune trajectories rather than cross-sectional snapshots, potentially relevant for ED decision-making before microbiological confirmation. From a precision-medicine perspective, functional immune testing may be most informative when interpreted within host-response endotypes, as molecular or clinical phenotypes differ in prognosis and may show differential treatment effects [23,40]. An indicative TARA low-response result in a C2-like phenotype may signal a high-risk state combining inflammation and impaired responsiveness, whereas the same result in C1 or C3 may carry a different meaning.

This study has several limitations. It was a single-center study with 152 patients, limiting power for mortality and subgroup analyses; the 24 h subset was smaller, and missing longitudinal data may have influenced trajectory characterization. The clustering was unsupervised and exploratory, the three-cluster solution requires external validation, and the panel may not capture endothelial injury, coagulation, metabolism, complement or transcriptomic programs. Survival analyses were event-limited and hypothesis-generating, and this observational design cannot determine whether TARA-guided management improves outcomes.

### 4.3. Potential Clinical Implications

The present study establishes a biological association and provides a rationale for prospective studies evaluating whether TARA-guided management improves clinical outcomes. Accordingly, TARA should not currently be used as a stand-alone tool for antibiotic decisions, ICU admission or immunomodulatory treatment. Its most plausible near-term role is as an adjunctive immune-stratification assay interpreted alongside clinical assessment, severity scores, conventional biomarkers, microbiological testing and measures of infection probability. Reduced inducible TNF-α responsive-ness may identify patients with immune dysfunction not apparent from physiological severity alone and could support closer reassessment or serial biological monitoring. Future prospective studies should determine whether incorporating TARA into clinical pathways improves treatment allocation, antibiotic stewardship or patient outcomes, and whether it can enrich trials of immune-restorative therapies.

### 4.4. Strengths and Limitations

The principal contribution of this study is demonstrating that a TARA rapid point-of-care functional immune assay independently identifies an early cytokine-low immune phenotype. By directly assessing immune responsiveness, it provides biological information complementary to conventional biomarkers and clinical severity scores, with the potential to enable real-time bedside immune phenotyping in the Emergency Department. Whether its integration into clinical decision-making improves patient outcomes requires prospective evaluation.

Strengths of this study include its prospective design, standardized early sampling, blinded clinical adjudication, independent derivation of cytokine-based variables, and use of a continuous LRCS. Internal bootstrap validation and an alternative two-cluster analysis further supported the robustness of the cytokine-low dimension.

Several limitations should be acknowledged. This single-center study included a moderate sample size, which may limit generalizability. As an exploratory unsupervised analysis, no formal sample-size calculation was performed, and inclusion required sufficient 4 h cytokine measurements, defined as the availability of at least 10 of the 12 analytes. This may have introduced selection bias by underrepresenting patients with very early deterioration, early transfer, or unavailable repeat sampling.

Fully adjusted models excluded seven patients without PCT measurements. Sensitivity analyses excluding PCT included the larger paired cohort and yielded consistent results, although multiple imputation or inverse-probability weighting was not performed.

Clustering was exploratory and based on k-means, which assumes discrete groups despite immune responses likely existing along overlapping biological continua. The modest silhouette coefficient reflected partial overlap between phenotypes. Bootstrap analyses showed good stability for the two largest clusters, whereas the third was less reproducible. The alternative two-cluster solution also identified a stable cytokine-low dimension, suggesting this biological signal is more robust than the precise boundaries of the three-cluster partition. External validation and comparison with complementary clustering methods remain necessary.

Finally, secondary analyses were not adjusted for multiple comparisons and should be considered exploratory. Clinical outcome analyses were limited by the number of events, and the observational design precludes causal inference. Interventional studies are needed to determine whether TARA-guided management improves patient outcomes.

## 5. Conclusions

In this exploratory ED cohort, an indicative TARA low-response status was independently associated with an early continuous cytokine-low dimension defined without reference to TARA or clinical outcomes. Bootstrap analyses supported the internal stability of the cytokine-low phenotype, although the specific three-cluster structure and phenotype-level clinical findings require external validation. TARA may provide a complementary functional readout of reduced ex vivo LPS-induced TNF-α responsiveness, but prospective studies are needed before it can be incorporated into clinical decision-making or treatment-selection pathways.

## Figures and Tables

**Table 1:** Overall characteristics according to the cytokine-derived immune phenotype assignment at 4 h. Data are median (IQR) for continuous variables and n/N (%) for categorical variables. *p* values were calculated using Kruskal–Wallis tests for continuous variables and chi-square tests for categorical variables, C1 denotes the cytokine-low phenotype, C2 the inflammatory/intermediate phenotype and C3 the IFN/Th17-high phenotype. ARB, angiotensin II receptor blocker; BMI, body mass index; CRP, C-reactive protein; ICU, intensive care unit; TARA, TNF-α release assay. Continuous variables were summarized among patients with available data. Binary variables show available denominators. ICU length of stay was summarized among patients with recorded ICU stay.

Variable	Overall (*N* = 152)	C1 Cytokine-Low (*N* = 56)	C2 Inflammatory/ Intermediate (*N* = 63)	C3 IFN/Th17-High (*N* = 33)	*p* Value
Demographics and anthropometrics					
Age, years	76 (63–84)	78 (65–87)	75 (62–84)	74 (67–81)	0.406
Female sex, n/N (%)	52/152 (34.2)	20/56 (35.7)	22/63 (34.9)	10/33 (30.3)	0.863
Height, cm	165.0 (157.2–170.8)	161.0 (156.0–169.0)	165.5 (160.0–171.2)	167.0 (158.0–170.0)	0.340
Weight, kg	72.0 (62.0–81.0)	70.0 (61.0–78.0)	73.0 (66.5–82.5)	72.0 (54.0–82.0)	0.518
BMI, kg/m^2^	26.0 (22.9–29.6)	27.5 (22.9–29.4)	26.1 (22.9–30.2)	24.9 (23.1–28.4)	0.789
Medical history					
Myocardial infarction, prior, n/N (%)	19/152 (12.5)	8/56 (14.3)	7/63 (11.1)	4/33 (12.1)	0.870
Heart failure, n/N (%)	30/152 (19.7)	10/56 (17.9)	12/63 (19.0)	8/33 (24.2)	0.753
Dementia, n/N (%)	36/152 (23.7)	10/56 (17.9)	15/63 (23.8)	11/33 (33.3)	0.253
Diabetes, n/N (%)	42/152 (27.6)	13/56 (23.2)	21/63 (33.3)	8/33 (24.2)	0.415
Chronic kidney disease, n/N (%)	35/152 (23.0)	11/56 (19.6)	11/63 (17.5)	13/33 (39.4)	0.040
Solid tumor malignancy, n/N (%)	29/152 (19.1)	12/56 (21.4)	9/63 (14.3)	8/33 (24.2)	0.426
Hematologic disease, n/N (%)	4/152 (2.6)	3/56 (5.4)	0/63 (0.0)	1/33 (3.0)	0.188
Liver disease, n/N (%)	12/152 (7.9)	4/56 (7.1)	2/63 (3.2)	6/33 (18.2)	0.034
Baseline medication					
Beta-blockers, n/N (%)	30/146 (20.5)	12/55 (21.8)	9/60 (15.0)	9/31 (29.0)	0.279
ACE inhibitors/ARBs, n/N (%)	40/145 (27.6)	12/55 (21.8)	20/59 (33.9)	8/31 (25.8)	0.343
Calcium-channel blockers, n/N (%)	8/142 (5.6)	2/54 (3.7)	3/57 (5.3)	3/31 (9.7)	0.510
Anticoagulants, n/N (%)	78/145 (53.8)	31/55 (56.4)	30/59 (50.8)	17/31 (54.8)	0.833
Statins, n/N (%)	55/146 (37.7)	20/55 (36.4)	24/60 (40.0)	11/31 (35.5)	0.886
Corticosteroids, n/N (%)	28/144 (19.4)	11/55 (20.0)	11/58 (19.0)	6/31 (19.4)	0.990
Clinical severity scores at presentation					
NEWS2 ≥ 5 at presentation, n/N (%)	94/152 (61.8)	30/56 (53.6)	36/63 (57.1)	28/33 (84.8)	0.008
qSOFA ≥ 2 at presentation, n/N (%)	26/152 (17.1)	8/56 (14.3)	12/63 (19.0)	6/33 (18.2)	0.775
Routine laboratory markers at presentation					
PCT ≥ 2 ng/mL at 0 h, n/N (%)	81/145 (55.9)	28/55 (50.9)	37/60 (61.7)	16/30 (53.3)	0.486
CRP ≥ 10 mg/dL at 0 h, n/N (%)	59/122 (48.4)	22/43 (51.2)	23/52 (44.2)	14/27 (51.9)	0.733
Lactate ≥ 18 mg/dL at 0 h, n/N (%)	63/112 (56.3)	13/37 (35.1)	31/46 (67.4)	19/29 (65.5)	0.007
TARA functional status					
Indicative TARA low-response result at 0 h, n/N (%)	96/148 (64.9)	43/55 (78.2)	39/60 (65.0)	14/33 (42.4)	0.003
Indicative TARA low-response result at 4 h, n/N (%)	97/148 (65.5)	41/55 (74.5)	41/60 (68.3)	15/33 (45.5)	0.018
Indicative TARA low-response result at 24 h, n/N (%)	68/97 (70.1)	32/37 (86.5)	22/35 (62.9)	14/25 (56.0)	0.018
Clinical outcomes					
Sepsis within 24 h, n/N (%)	119/152 (78.3)	44/56 (78.6)	49/63 (77.8)	26/33 (78.8)	0.991
Hospital length of stay, days	7.0 (3.0–14.0)	6.5 (3.3–12.0)	7.0 (2.0–15.0)	7.0 (4.0–14.2)	0.907
ICU length of stay, days	5.0 (2.5–11.5)	4.4 (3.3–16.5)	6.0 (3.0–13.0)	4.0 (2.2–8.8)	0.874

## Data Availability

The original contributions presented in this study are included in the article/Supplementary Materials. Further inquiries can be directed to the corresponding authors. The analysis code reported in this study has been deposited in the institutional CORA Research Data Repository, managed by the Consortium of University Services of Catalonia (CSUC), under the following DOI: https://doi.org/10.34810/DATA3601.

## Supplementary Materials

The following supporting information can be downloaded at: https://www.mdpi.com/article/10.3390/medsci14040465/s1, Supplementary Methods; Figure S1: TARA components and point-of-care workflow; Figure S2: Cluster-number sensitivity analysis; Figure S3: Bootstrap validation of the three-cluster solution; Figure S4: Patient flow for the 4 h cytokine analysis; Figure S5: Exploratory competing-risk analysis by LRCS; Figure S6: Sensitivity analysis using the k = 2 clustering solution; Table S1: Comparison of included and non-included patients; Table S2: Patient characteristics under the k = 2 clustering solution; Table S3: Exploratory analysis by pathogen Gram status.
