# Supplementary material for: Early Identification of a Cytokine-Low Immune Phenotype in Suspected Sepsis Using a Point-of-Care Whole-Blood TNF-α Release Assay
Source: Med Sci (Basel). 2026 Aug 8;14(4):465. doi: 10.3390/medsci14040465 (PMC13515652; doi:10.3390/medsci14040465)
Supplement: Supplementary file 1 [file medsci-14-00465-s001.zip › medsci-4459381-supplementary.pdf]

Supplemental Data File:

Supplementary Methods

Supplementary Materials and Methods: TARA functional immune assay

The TNF-α Release Assay (TARA; SeptiLoop®, Loop Diagnostics, Spain) is an ex vivo whole-blood functional immune assay designed to assess inducible TNF-α release after standardized lipopolysaccharide (LPS) stimulation. The assay evaluates the functional capacity of circulating innate immune cells to respond to an endotoxin challenge. Therefore, unlike direct plasma cytokine measurements, TARA does not quantify basal circulating TNF-α, but measures stimulated TNF-α release after ex vivo activation of whole blood.

Fresh whole blood was collected in lithium-heparin tubes and processed according to the manufacturer's instructions. Briefly, 1 mL of whole blood was mixed with the assay reagents containing purified LPS and incubated in a controlled dry heat block at 37 °C for approximately 2.5 h. After stimulation, the assay device was activated and stimulated TNF-α release was assessed using a semiquantitative lateral-flow immunoassay. Results were interpreted within the predefined reading window, approximately 19–21 min after lateral-flow activation. The total time from blood collection to result availability was approximately 3 h.

TARA results were interpreted according to the predefined qualitative assay classification. Samples showing preserved LPS-induced TNF-α release were classified as preserved response. Samples showing reduced or undetectable LPS-induced TNF-α release were classified as indicative low-response, compatible with impaired innate immune responsiveness. An indicative low-response TARA result was predefined as stimulated TNF-α release below 250 pg/mL, corresponding to the validated analytical limit of detection of the assay. Although the lateral-flow band intensity provides a semiquantitative estimate of

stimulated TNF-α release, only the predefined qualitative classification, indicative low-response versus preserved response, was used for the clinical analyses.

The analytical limit of detection of SeptiLoop® was defined as the lowest TNF-α concentration at which at least 95% of replicate tests, corresponding to ≥19 of 20 replicates, yielded a positive result. Serial TNF-α dilutions were tested in the clinical matrix across replicate devices. The lowest concentration meeting this predefined criterion was 250 pg/mL, at which TNF-α was detected in 100% of replicates. At the next lower concentration, 200 pg/mL, TNF-α was detected in 85% of replicates and therefore did not meet the predefined acceptance criterion. Accordingly, the analytical limit of detection of the SeptiLoop® device was established at 250 pg/mL.

The analytical performance of TARA had been established before the present clinical analyses. Analytical validation included assessment of limit of detection, specimen stability, interference, precision, reproducibility, robustness, ruggedness and hook effect. Whole-blood specimen stability was demonstrated for up to 16 h after collection under validated handling conditions. Precision studies showed high repeatability and reproducibility across operators, testing sites and device lots, supporting the robustness of the qualitative interpretation used in this study.

All TARA procedures were performed by trained personnel following the manufacturer's instructions and predefined validity criteria. Tests were considered valid only when the lateral-flow control signal and sample migration were adequate. Invalid tests, including failed migration or absent control signal, were excluded from analysis or repeated when sufficient sample was available. Personnel interpreting TARA results were blinded to multiplex cytokine measurements, clinical severity scores, Sepsis-3 classification and hospital outcomes.

For the purpose of the present study, TARA was used as a functional immune stratification assay to identify patients with reduced ex vivo LPS-induced TNF-α release. The assay result was not used to guide clinical management, treatment allocation or outcome adjudication.

Definition of the primary cytokine analysis cohort

The primary cytokine analysis was conducted in patients with sufficient cytokine data at 4 h to calculate the Low-Response Cytokine Score and define cytokine-derived immune phenotypes. Patients were eligible for this analysis when at least 10 of the 12 cytokines were available at 4 h. The final analysis cohort comprised 152 patients.

Of these, 148 had paired LRCS-4h and TARA results at presentation, and 148 had paired LRCS-4h and TARA results at 4 h. Complete-case cohorts for the clinically adjusted models included 148 patients for both the presentation TARA and 4 h TARA analyses. Complete-case cohorts for the fully adjusted models including PCT included 141 patients for both analyses. The score was constructed independently of TARA, clinical variables, microbiology and hospital outcomes.

For each of the 12 cytokines, the 4 h concentration was transformed as:

$$\log_{10}(\text{concentration} + 1)$$

Patients with at least 10 of 12 cytokines available at 4 h were included. Isolated missing cytokine values were imputed using the median value of the corresponding cytokine in the 4 h reference cohort. Each cytokine was then standardised within the reference cohort:

$$z_j = \frac{\log_{10}(x_j + 1) - \mu_j}{\sigma_j}$$

where j denotes each cytokine, and μ_j and σ_j represent the mean and standard deviation of the transformed cytokine in the 4 h reference cohort after isolated-value imputation.

A raw low-response score was calculated as the negative mean of the 12 standardized cytokine values:

$$LRCS_{\text{raw}} = -\frac{1}{12}\sum_{j=1}^{12} z_j$$

The final LRCS-4h was standardised to a mean of 0 and a standard deviation of 1 in the 4 h reference cohort:

$$LRCS\text{-}4h = \frac{LRCS_{\text{raw}} - \mu_{\text{LRCS raw}}}{\sigma_{\text{LRCS raw}}}$$

Higher LRCS-4h values therefore indicate globally lower circulating cytokine concentrations. Across the 152-patient cohort, 0.66% of individual cytokine values at 4 h required imputation. No LRCS cut-off was derived in this cohort, and the score was analyzed as a continuous variable throughout the primary analyses.

Primary association between TARA and the cytokine-low phenotype

The primary analysis evaluated whether an indicative low-response TARA result measured at 4 h was associated with higher LRCS-4h values. This analysis was selected as primary because TARA and LRCS were measured within the same early biological time window.

The association between TARA status and LRCS-4h was evaluated using ordinary least squares linear regression with heteroskedasticity-consistent HC3 robust standard errors. TARA status was coded as a binary variable, with indicative low-response results coded as 1 and preserved-response results as "non-indicative" coded as 0. Regression coefficients were expressed in standard deviation units of LRCS-4h. Three hierarchical models were evaluated: Unadjusted model: TARA status only; Clinically adjusted model: TARA status, age, Charlson comorbidity index, baseline NEWS2, time from symptom onset to baseline

sampling and baseline corticosteroid treatment; and, Clinically adjusted + PCT model: the clinically adjusted model with additional inclusion of log10(PCT + 1).

Internal validation of cluster stability

Cluster stability was assessed using 1,000 non-parametric bootstrap resamples. In each iteration, patients were sampled with replacement and the complete preprocessing and clustering pipeline was repeated, including log10(concentration + 1) transformation, median imputation of isolated missing cytokine values, standardization and k-means clustering with k=3, k-means++ initialization and 100 random initializations. Overall partition agreement was quantified using the adjusted Rand index. Cluster-specific stability was evaluated using the maximum Jaccard similarity between each original cluster and the most similar bootstrap-derived cluster among the unique observations represented in each bootstrap sample. Higher values indicated greater reproducibility of phenotype membership.

TARA assay procedure

Fresh lithium-heparin whole blood was used according to the manufacturer's instructions. One milliliter of whole blood was incubated with purified LPS on a controlled dry heat block, and stimulated TNF-α release was subsequently assessed using a semiquantitative lateral-flow immunoassay after approximately 2.5 h of stimulation.

Plasma cytokine panel

The cytokine panel comprised 12 analytes: interferon-γ (IFN-γ); interleukin-10 (IL-10); interleukin-17A (IL-17A); interleukin-1β (IL-1β); interleukin-1 receptor antagonist (IL-1RA); interleukin-6 (IL-6); interleukin-7 (IL-7); interleukin-8 (IL-8); monocyte

chemoattractant protein-1 (MCP-1); monocyte chemoattractant protein-3 (MCP-3); tumor necrosis factor-α (TNF-α); tumor necrosis factor receptor I (TNF-RI).

Complete-case analysis was used for each model. The model including clinical covariates and PCT constituted the fully adjusted primary model.

A key secondary analysis evaluated the association between TARA measured at presentation and LRCS-4h using the same model structure. This analysis assessed whether an early functional low-response result at presentation was associated with the cytokine-low phenotype observed at 4 h.

Clustering procedure

K-means solutions with k=2 to k=5 clusters were examined using silhouette coefficients calculated in the original standardized cytokine feature space. Clustering was performed using k-means++ initialization with 100 random initializations and a fixed random seed to ensure reproducibility.

Sensitivity analyses

Sensitivity analyses were performed to evaluate the robustness of the primary association between TARA and LRCS 4h. These included robust linear regression based on a Huber M estimator and reduced clinical adjustment models including age, Charlson comorbidity index and baseline NEWS2, with or without log10(PCT + 1). Collinearity among covariates was assessed using variance inflation factors.

Distribution of TARA results across immune phenotypes

To complement the continuous LRCS analysis, the distribution of indicative TARA results at 4 h was compared across C1, C2 and C3. The number and proportion of patients with indicative TARA results were calculated using available TARA results at each time point as denominators.

Comparisons of TARA proportions across the three immune phenotypes were performed using Fisher's exact test with Monte Carlo estimation for contingency tables where appropriate. Analyses of TARA at presentation and 24 h across immune phenotypes were considered secondary or supplementary longitudinal analyses.

Phenotype level logistic regression

Additional phenotype level analyses were performed to support the interpretation of Figure 1. Logistic regression models estimated adjusted odds ratios for predefined contrasts across TARA time points. The first contrast evaluated whether an indicative TARA result was associated with membership in the C1 cytokine low phenotype. The second contrast evaluated whether a preserved TARA response was associated with membership in the C3 IFN/Th17 high phenotype.

Models used a covariate structure consistent with the panel E/F odds-ratio analyses when data availability allowed: age, sex, Charlson comorbidity index, baseline SOFA and log10(PCT + 1). Odds ratios were reported with 95% confidence intervals and p values. These analyses were secondary and should be interpreted as phenotype level supportive analyses rather than independent confirmatory endpoints.

Longitudinal biological characterization of immune phenotypes

Phenotype membership was fixed according to the 4 h clustering solution. Cytokine measurements obtained at presentation and 24 h were not used to redefine phenotypes and were analyzed only for longitudinal biological characterization.

For longitudinal visualization, cytokine values measured at 0 h and 24 h were transformed and projected using the orientation and standardization reference established from the 4 h cytokine matrix. A projected low-response score was generated at 0 h and 24 h using the

same directionality as LRCS-4h, such that higher values represented globally lower circulating cytokine concentrations.

This projected longitudinal score was used as a descriptive secondary analysis to characterise temporal biological patterns. It was not considered a validated endpoint at time points other than 4 h and was not used to derive new phenotype assignments or prognostic thresholds.

Longitudinal profiles of representative cytokines were described according to fixed immune phenotype membership. IL-6, IL-1RA and MCP-1 were selected to represent the inflammatory/intermediate profile of C2, whereas IFN-γ, MCP-3 and IL-17A were selected to represent the IFN/Th17-high profile of C3.

Clinical presentation, microbiological documentation and hospital course

Clinical and microbiological characteristics were compared across cytokine derived immune phenotypes. Variables included suspected infection focus, any documented organism, positive blood culture, shock, vasopressor use, ICU admission, in hospital death, hospital length of stay and ICU length of stay when applicable.

Recorded microbiological documentation was derived from the available organism field in the dataset. Absence of a documented organism was interpreted as absence of a recorded organism and not as confirmed microbiological negativity. Positive blood culture proportions were calculated among patients with available blood culture results. Clinical outcome analyses were descriptive and exploratory. They were not used for phenotype derivation, LRCS construction or the primary TARA association analysis.

Exploratory mortality pathway analysis

Because the primary biological analyses indicated that the cytokine low phenotype was enriched in indicative TARA low response results but was not the phenotype with the highest recorded shock or ICU admission, exploratory analyses evaluated the relationship between phenotype membership and in hospital mortality pathway.

Patients were classified as alive or discharged, death after recorded ICU admission, or death without recorded ICU admission. These analyses were considered hypothesis generating. ICU eligibility, treatment limitation decisions, goals of care discussions and palliative care status were not available in the dataset; therefore, death without recorded ICU admission should not be interpreted as evidence of inadequate escalation of care.

Exploratory competing-risk analyses

Exploratory competing risk analyses were conducted using a 4 h landmark framework. Patients who experienced a terminal event before the 4 h landmark were excluded from these analyses. In hospital death and discharge alive were treated as mutually competing terminal outcomes.

Cumulative incidence functions were estimated non parametrically using the Aalen Johansson approach according to tertiles of LRCS 4h and according to cytokine derived immune phenotype. These analyses were exploratory and were not designed or powered to establish prognostic utility of LRCS, TARA or immune phenotype membership.

Statistical analysis

Statistical analyses were performed using Python version 3.13.5. Data management and variable transformation were conducted using pandas version 2.2.3 and NumPy version 2.3.5. Principal component analysis and k means clustering were implemented using scikit learn

version 1.8.0. Regression analyses were implemented using statsmodels version 0.14.6. Figures were generated using Matplotlib version 3.10.8 and exported in vector PDF format.
Continuous variables were summarised as medians with interquartile ranges for descriptive tables and as means with standard errors of the mean for longitudinal graphical representations. Comparisons across cytokine derived immune phenotypes were performed using Kruskal Wallis tests for continuous variables and Fisher exact tests or chi square tests for categorical variables, with Monte Carlo estimation when appropriate. Pairwise comparisons between TARA preserved response and indicative low response groups were performed using two-sided Mann Whitney U tests.

The primary association between indicative TARA low response status and LRCS 4h was evaluated using ordinary least squares linear regression with HC3 robust standard errors. Regression coefficients were expressed in standard deviation units of LRCS 4h. Sensitivity analyses used robust linear regression with a Huber M estimator. All tests were two sided, and p values below 0.05 were considered statistically significant. No adjustment for multiplicity was applied to secondary or exploratory figure level comparisons, which should therefore be interpreted as descriptive or hypothesis generating.

Supplementary Figure S1 | Components and point-of-care workflow of TARA assay (SeptiLoop®, novel CE marked device under IVDR class C).

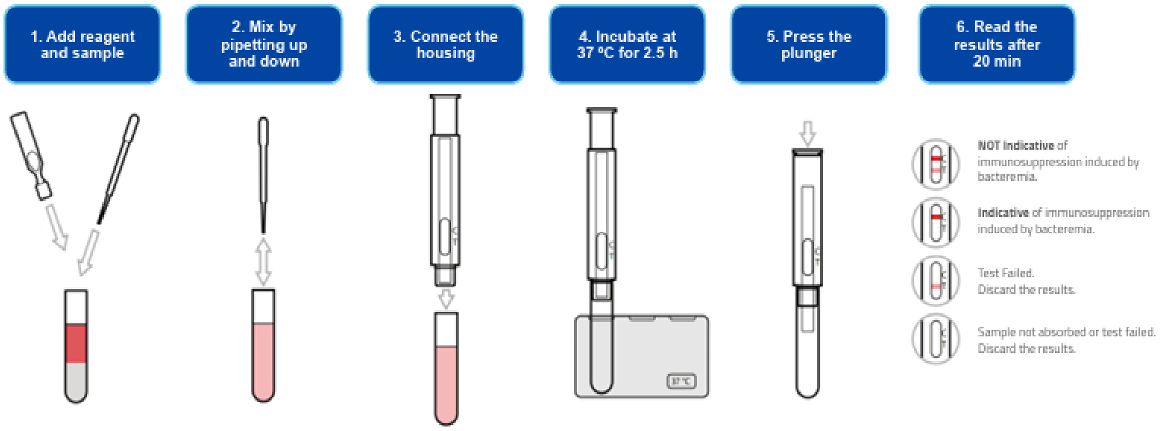

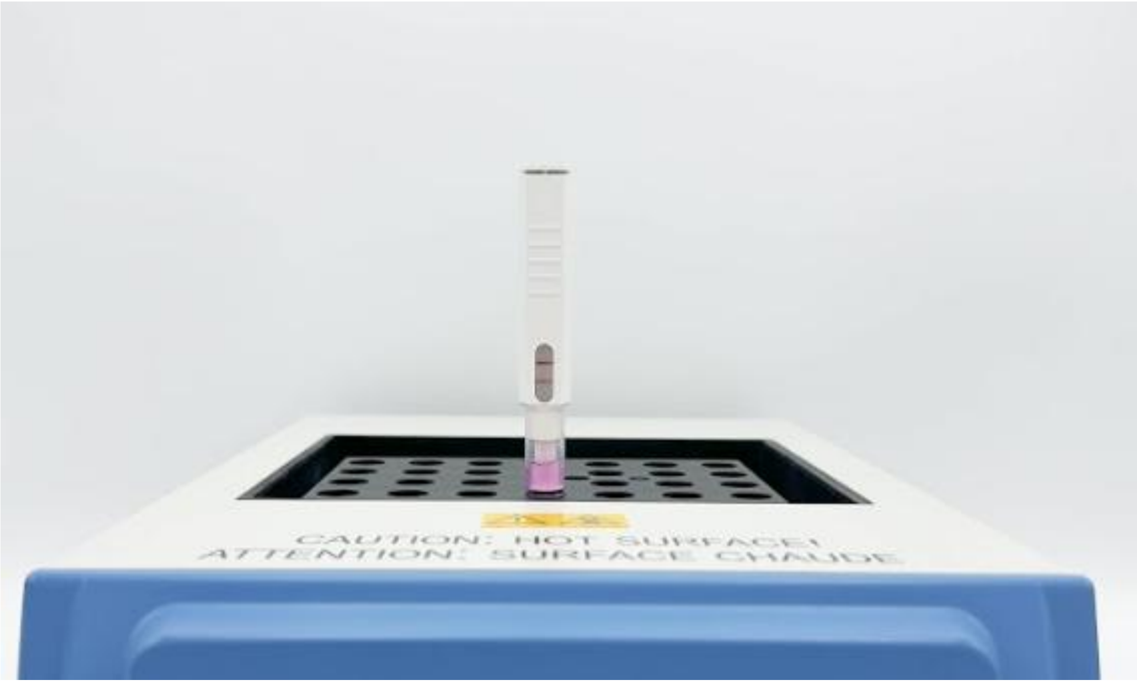

Supplementary Figure S2 | Cluster-number sensitivity analysis for cytokine clustering at 4 h.
A, Silhouette scores for solutions with k=2 to k=5 clusters, calculated directly on the standardized 12-cytokine matrix at 4 h. Clustering was evaluated in the original cytokine feature space without using dimensionality-reduction coordinates for phenotype definition. k=3 was selected as the primary solution; the k=2 solution is reported in Supplementary Table 2 to demonstrate stability of the cytokine-low phenotype across cluster number.

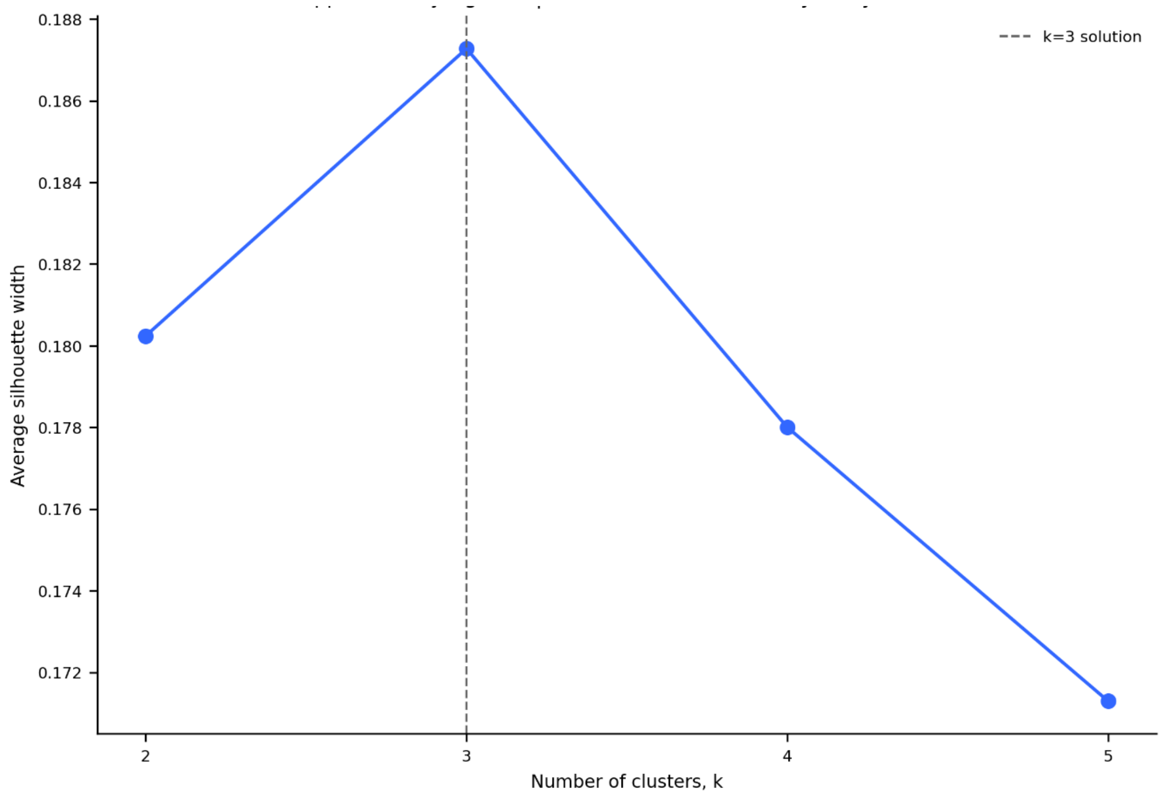

Supplementary Figure S3 | Bootstrap internal validation of the three-cluster cytokine solution. Distribution of the overall adjusted Rand index and cluster-specific Jaccard similarity coefficients across 1,000 non-parametric bootstrap resamples. In each resample, the complete cytokine preprocessing and k-means clustering pipeline was repeated. Cluster-specific Jaccard similarity was calculated between each original phenotype and the most similar bootstrap-derived phenotype among unique in-bootstrap observations. Boxes represent the interquartile range, central solid lines indicate medians, dashed lines indicate means, and whiskers represent the 2.5th and 97.5th percentiles.

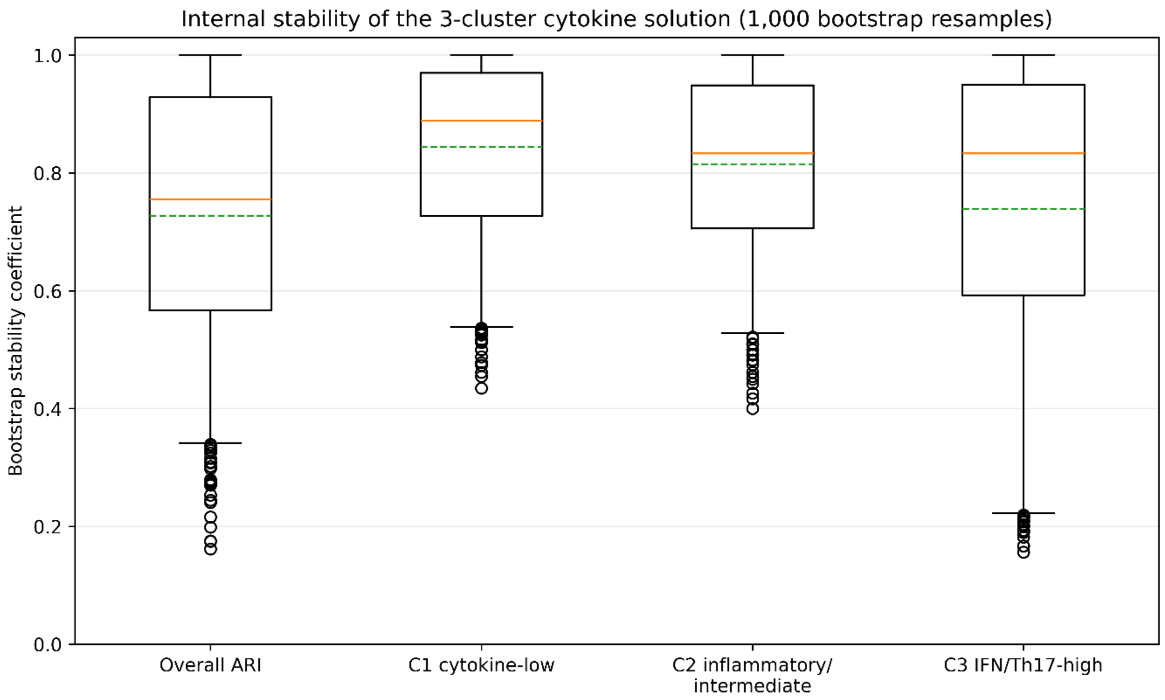

Supplementary Figure S4 | Patient flow for the 4 h cytokine analysis.
Flow diagram showing the available cytokine-clinical dataset, the cohort with sufficient cytokine data to derive LRCS at 4 h, the paired TARA analysis cohorts at presentation and 4 h, and the complete-case cohorts included in the fully adjusted regression models. LRCS, Low-Response Cytokine Score; PCT, procalcitonin; TARA, TNF-α Release Assay.

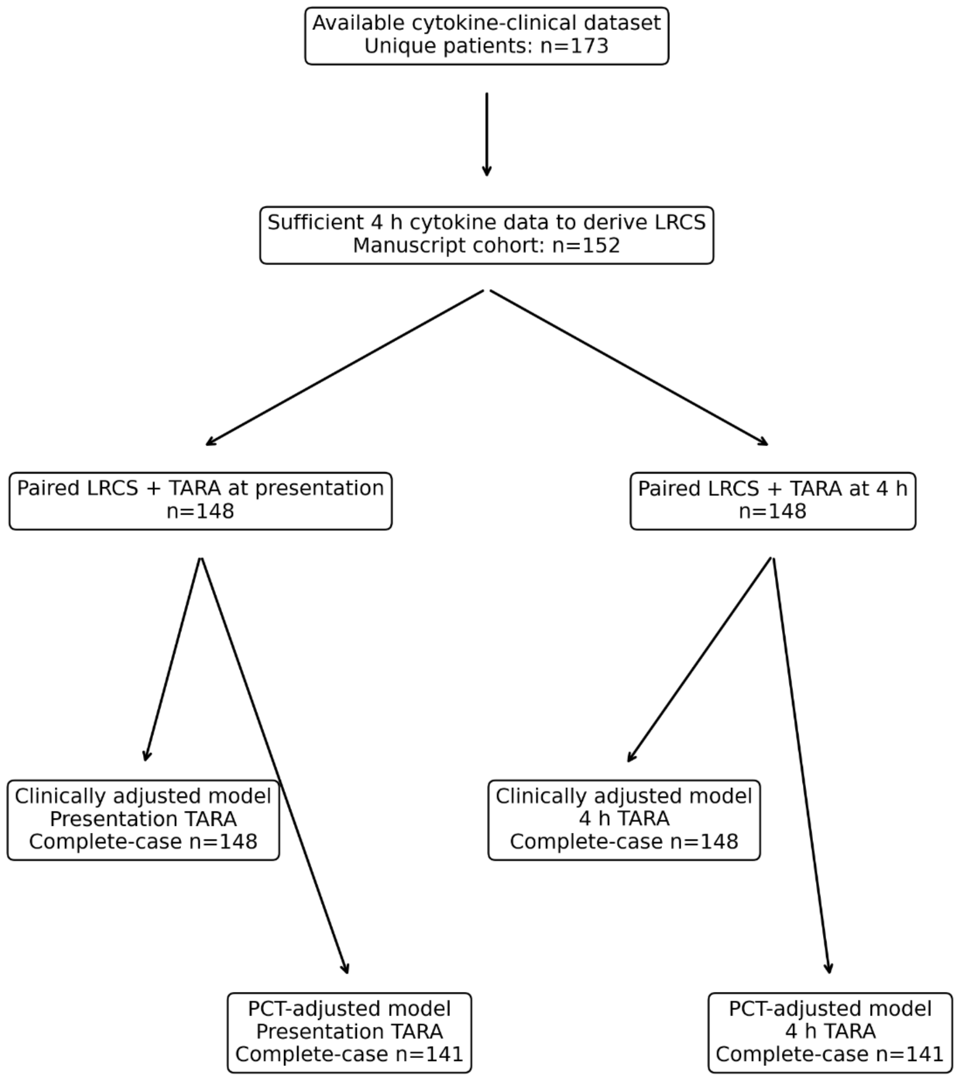

Supplementary Figure S5 | Exploratory competing-risk analyses by LRCS. A–B, Cumulative incidence functions for in-hospital death and discharge alive according to tertiles of LRCS, using a 4 h landmark framework and treating discharge alive and in-hospital death as mutually competing terminal outcomes. These analyses were exploratory and were not designed or powered to establish prognostic utility. LRCS, Low-Response Cytokine Score.

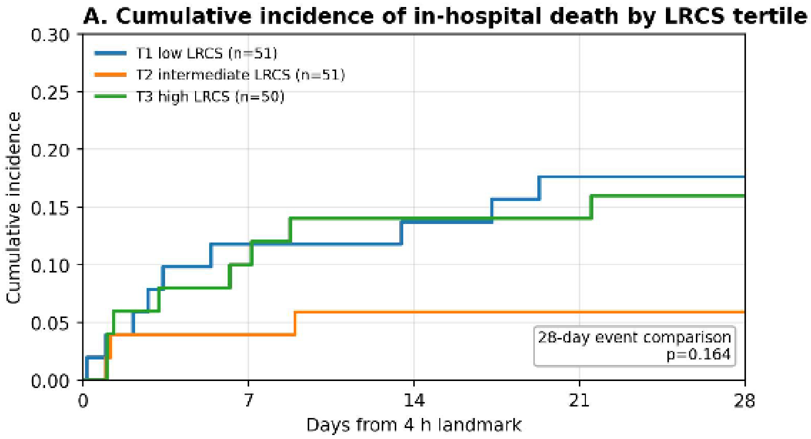

At risk	0	7	14	21	28
T1 low LRCS	51	32	18	9	7
T2 intermediate LRCS	51	31	14	12	9
T3 high LRCS	50	26	11	6	3

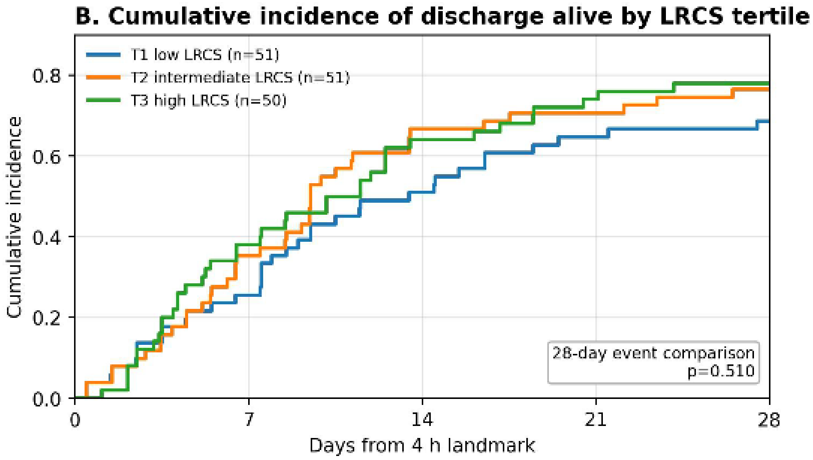

At risk	0	7	14	21	28
T1 low LRCS	51	32	18	9	7
T2 intermediate LRCS	51	31	14	12	9
T3 high LRCS	50	26	11	6	3

Supplementary Figure S6 | Sensitivity analysis under the alternative k=2 cytokine-clustering solution. The k=2 solution identified C1 cytokine-low (n=65) and C2 cytokine-activated (n=87). **A**, PCA visualization of the two phenotypes; crosses indicate cluster centroids. PCA was used only for visualization. **B**, TARA functional status at 4 h projected onto the same PCA space. **C**, LRCS-4h according to TARA status at presentation and 4 h. **D**, linear-regression estimates for the association of indicative TARA status at presentation or 4 h with LRCS-4h. Clinically adjusted models included age, Charlson comorbidity index, baseline NEWS2, symptom duration, and baseline corticosteroid treatment; PCT-adjusted models additionally included log10(PCT+1). Points represent β coefficients and horizontal bars indicate 95% confidence intervals. **E**, LRCS-4h across the two cytokine-derived phenotypes, with the proportion of indicative TARA results overlaid. LRCS, Low-Response Cytokine Score; NEWS2, National Early Warning Score 2; PCA, principal component analysis; PCT, procalcitonin; TARA, TNF-α Release Assay

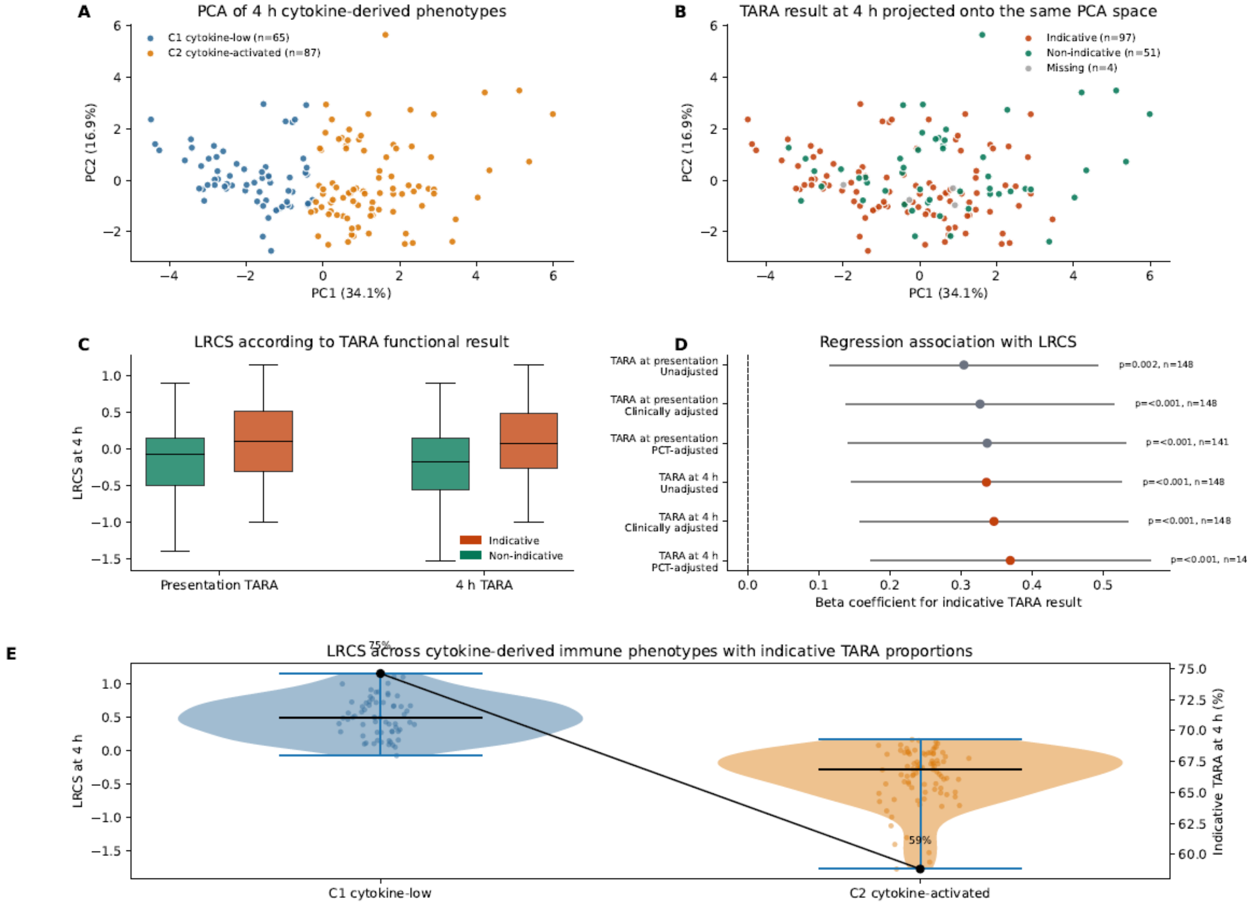

Supplementary Table S1. Comparison of patients included and not included in the 4 h cytokine analysis. Data are median (interquartile range) unless otherwise indicated. Continuous variables were compared using two-sided Mann–Whitney U tests and categorical variables using Fisher's exact test. The comparisons were descriptive and were not adjusted for multiplicity.

**Variable**	**Included cohort (N=152)**	**Not included (N=21)**	**Available N included**	**Available N not included**	**P value**	**Test**
Age, years	76.5 (63.0–84.2)	77.0 (67.0–85.0)	152	21	0,957	Mann–Whitney U
NEWS2 at presentation	5.0 (4.0–7.2)	6.0 (4.0–8.0)	152	21	0,473	Mann–Whitney U
qSOFA at presentation	1.0 (0.0–1.0)	1.0 (0.0–1.0)	152	20	0,662	Mann–Whitney U
SOFA at presentation	3.0 (1.8–4.0)	2.0 (2.0–5.0)	152	21	0,877	Mann–Whitney U
Shock, n/N (%)	16/152 (10.5%)	3/21 (14.3%)	152	21	0,707	Fisher exact
In-hospital death, n/N (%)	25/152 (16.4%)	4/21 (19.0%)	152	21	0,758	Fisher exact

Data are median (IQR) unless otherwise indicated. Continuous variables were compared using two-sided Mann–Whitney U tests; the effect size is rank-biserial correlation (included minus not included). Binary variables were compared using Fisher's exact test; the effect size is the odds ratio with 95% CI. No multiplicity adjustment was applied because this table assesses cohort comparability descriptively.

Supplementary table S2. Sensitivity analysis: patient characteristics under the k=2 clustering solution, in which the inflammatory/intermediate (C2) and IFN/Th17-high (C3) phenotypes merge into a single cytokine-activated group while the cytokine-low (C1) phenotype is preserved. Overall characteristics according to the cytokine-derived immune phenotype assignment at 4 h (k=2 solution).

Characteristic	Total N=152	C1 cytokine-low N=65	C2 cytokine-activated N=87	Global p
Demographics and comorbidity				
Age, years	76.5 (63.0-84.2)	78.0 (65.0-85.0)	75.0 (62.5-83.0)	0.381
Male sex	100/152 (65.8%)	40/65 (61.5%)	60/87 (69.0%)	0.389
Charlson comorbidity index	5.0 (4.0-7.0)	5.0 (4.0-7.0)	5.0 (4.0-7.0)	0.476
Clinical presentation at baseline				
Infection focus (available n=149)				0.258
Respiratory	66/149 (44.3%)	35/63 (55.6%)	31/86 (36.0%)	
Urinary	42/149 (28.2%)	14/63 (22.2%)	28/86 (32.6%)	
Abdominal	22/149 (14.8%)	8/63 (12.7%)	14/86 (16.3%)	
Skin and soft tissue	7/149 (4.7%)	1/63 (1.6%)	6/86 (7.0%)	
Unknown	6/149 (4.0%)	2/63 (3.2%)	4/86 (4.7%)	
Osteoarticular	3/149 (2.0%)	2/63 (3.2%)	1/86 (1.2%)	
CNS	2/149 (1.3%)	1/63 (1.6%)	1/86 (1.2%)	
Endocarditis	1/149 (0.7%)	0/63 (0.0%)	1/86 (1.2%)	
PCT, ng/mL	3.8 (0.7-8.5)	1.7 (0.5-8.0)	4.5 (0.9-8.5)	0.131
CRP, mg/dL	9.4 (3.4-24.0)	9.1 (3.0-25.4)	9.5 (3.9-22.5)	1.000
Lactate, mg/dL	20.4 (12.7-29.4)	15.6 (11.3-22.2)	24.1 (14.3-32.8)	<0.001
Leukocytes, ×10^9/L	12.3 (8.2-18.1)	12.4 (8.3-18.1)	12.2 (8.1-17.4)	0.978
Absolute neutrophil count, ×10^9/L	10.6 (6.5-15.7)	9.8 (6.3-15.6)	10.8 (6.6-15.8)	0.738
Absolute lymphocyte count, ×10^9/L	0.8 (0.4-1.3)	0.9 (0.5-1.4)	0.7 (0.3-1.1)	0.044
Neutrophil-to-lymphocyte ratio	12.8 (7.5-22.1)	10.7 (4.9-18.6)	14.6 (8.8-24.1)	0.027
SOFA score	3.0 (1.8-4.0)	3.0 (1.0-4.0)	3.0 (2.0-4.5)	0.114
APACHE II score	11.0 (8.0-15.0)	11.0 (8.0-15.0)	11.0 (8.0-15.0)	0.773
NEWS2 score	5.0 (4.0-7.2)	5.0 (3.0-7.0)	6.0 (4.0-9.0)	0.022
qSOFA score	1.0 (0.0-1.0)	1.0 (0.0-1.0)	1.0 (1.0-1.0)	0.052
Baseline management and immune-response confounders				
Corticosteroids at baseline	28/144 (19.4%)	12/63 (19.0%)	16/81 (19.8%)	1.000
Intravenous fluids at baseline	119/151 (78.8%)	52/65 (80.0%)	67/86 (77.9%)	0.842
Adequate antibiotics at baseline	131/134 (97.8%)	56/56 (100.0%)	75/78 (96.2%)	0.265
Biomarker and microbiology results				
TARA indicative low response at 0 h	96/148 (64.9%)	48/64 (75.0%)	48/84 (57.1%)	0.026
TARA indicative low response at 4 h	97/148 (65.5%)	47/63 (74.6%)	50/85 (58.8%)	0.055
TARA indicative low response at 24 h	68/97 (70.1%)	36/44 (81.8%)	32/53 (60.4%)	0.027
Exploratory hospital course and outcomes				
Clinical diagnosis of sepsis-3	119/152 (78.3%)	51/65 (78.5%)	68/87 (78.2%)	1.000
Shock	16/152 (10.5%)	1/65 (1.5%)	15/87 (17.2%)	0.001
Recorded ICU admission	25/152 (16.4%)	5/65 (7.7%)	20/87 (23.0%)	0.014
In-hospital death	25/152 (16.4%)	11/65 (16.9%)	14/87 (16.1%)	1.000
Time from symptom onset to baseline sample, days	2.0 (0.0-4.0)	2.0 (1.0-4.0)	2.0 (0.0-3.0)	0.334
Hospital length of stay, days	8.7 (3.7-16.3)	7.6 (4.1-12.7)	9.6 (3.7-16.7)	0.359

Values are median (IQR) or n/N (%), as applicable. Global p values were calculated using Mann-Whitney U tests for continuous variables and Fisher exact tests or chi-square/Monte Carlo tests for categorical variables. Immune phenotypes were defined by direct k-means clustering of the log10(x+1)-transformed and z-standardised 12-cytokine matrix at 4 h. In the 152-patient cohort, the k=2 C1 cytokine-low group largely overlapped with k=3 C1 (56/65, 86.2%), whereas the k=2 C2 cytokine-activated group comprised mainly k=3 C2 (59/87, 67.8%) and k=3 C3 (28/87, 32.2%).

Supplementary table S3. Exploratory analysis according to documented pathogen Gram status. Data are presented as n/N (%) or median (interquartile range). Categorical variables were compared using Fisher's exact test and continuous variables using two-sided Mann–Whitney U tests. The global phenotype-distribution p value was estimated using Fisher's exact test with Monte Carlo resampling. Analyses were exploratory and were not adjusted for multiplicity.

**Variable**	**Gram-negative (N=40)**	**Gram-positive (N=13)**	**Effect size**	**P value**	**Test**
Indicative TARA low response at presentation, n/N (%)	21/40 (52.5%)	7/13 (53.8%)	1.06 (0.30–3.70)	1,000	Fisher exact
Indicative TARA low response at 4 h, n/N (%)	19/39 (48.7%)	7/13 (53.8%)	1.23 (0.35–4.32)	1,000	Fisher exact
LRCS at 4 h, median (IQR)	-0.18 (-1.00–0.17)	-0.46 (-0.82–(-0.06))	-0.015	0,942	Mann–Whitney U
Circulating TNF-α at 4 h, pg/mL, median (IQR)	9.11 (5.37–20.85)	14.22 (6.82–42.34)	-0.169	0,369	Mann–Whitney U
C1 cytokine-low, n/N (%)	8/40 (20.0%)	3/13 (23.1%)		0,760	Fisher exact, Monte Carlo
C2 inflammatory/intermediate, n/N (%)	23/40 (57.5%)	6/13 (46.2%)			
C3 IFN/Th17-high, n/N (%)	9/40 (22.5%)	4/13 (30.8%)			
